# The Glyoxylate Cycle Is Involved in White-Opaque Switching in *Candida albicans*

**DOI:** 10.3390/jof7070502

**Published:** 2021-06-24

**Authors:** Susana Hidalgo Vico, Daniel Prieto, Rebeca Alonso Monge, Elvira Román, Jesús Pla

**Affiliations:** Departamento de Microbiología y Parasitología-IRYCIS, Facultad de Farmacia, Universidad Complutense de Madrid, Avda. Ramón y Cajal s/n, 28040 Madrid, Spain; shvico@ucm.es (S.H.V.); adprieto@ucm.es (D.P.); realonso@ucm.es (R.A.M.)

**Keywords:** commensalism, glyoxylate cycle, **wo** switching, fungal pathogenesis, epigenetics, opaque cells, oxidative stress

## Abstract

*Candida albicans* is a commensal yeast that inhabits the gastrointestinal tract of humans. The master regulator of the white-opaque transition *WOR1* has been implicated in the adaptation to this commensal status. A proteomic analysis of cells overexpressing this transcription factor (*WOR1^OE^*) suggested an altered metabolism of carbon sources and a phenotypic analysis confirmed this alteration. The *WOR1^OE^* cells are deficient in using trehalose and xylose and are unable to use 2C sources, which is consistent with a reduction in the amount of Icl1, the isocitrate lyase enzyme. The *icl1Δ/Δ* mutants overexpressing *WOR1* are deficient in the production of phloxine B positive cells, a main characteristic of opaque cells, a phenotype also observed in mating type hemizygous *mtla1Δ icl1Δ/Δ* cells, suggesting the involvement of Icl1 in the adaptation to the commensal state. In fact, *icl1Δ/Δ* cells have reduced fitness in mouse gastrointestinal tract as compared with essentially isogenic heterozygous *ICL1*/*icl1Δ,* but overproduction of *WOR1* in an *icl1Δ/Δ* mutant does not restore fitness. These results implicate the glyoxylate shunt in the adaptation to commensalism of *C. albicans* by mechanisms that are partially independent of *WOR1*.

## 1. Introduction

*Candida albicans* is a commensal yeast that inhabits the gastrointestinal tract of healthy people, where it behaves as a harmless commensal. When host defenses are altered, *C. albicans* can translocate through the gut epithelium and disseminate via the blood stream, causing severe systemic diseases with as much as 50% mortality [1,2,3]. Previously, several virulence factors have been identified in this fungus using an acute systemic infection model in mice [4]. However, in recent years, the popularization of models of colonization of the mammalian gastrointestinal tract [5] has allowed the identification of factors promoting the establishment of the fungus in this location. Inhibition of colonization may promote the eradication (or, at least, the control) of fungal populations in this niche and could be an effective prophylactic approach for the prevention of endogenous candidiasis in immunosuppressed patients [6,7,8,9]. Several genes have been shown to be involved in colonization (see [10,11,12] for recent reviews) and their nature is diverse as they regulate the metabolic status of the fungus and encode components of signaling pathways and/or morphogenetic transitions [13,14,15,16,17,18,19].

The white opaque transition (**wo**) is an environmentally regulated epigenetic program that produces opaque (mating competent) cells from homozygous **a**/**a** and α/α diploids, therefore, allowing mating and subsequent tetraploid formation (see [20] for a review). Opaque cells differ from white cells in that they are more elongated, positive to phloxine B staining, and having a distinct transcriptional program [21,22,23]. The **wo** conversion has important biological consequences, as opaque cells colonize more readily the skin of mammals [24] but are less virulent as compared with white cells in a mouse model of systemic infection [25], consistent with their unstable phenotype at high (37 °C) temperatures. Wor1 is the master regulator of the **wo** transition; deletion of *WOR1* blocks the conversion to the opaque phase and its expression is repressed in heterozygous mating type white cells by the action of two homeodomain proteins (**a**1/α2) [26,27,28,29]. Other **wo** regulators have been identified among previously identified opaque-enriched genes [22,23] by in silico selection of transcriptional regulators [30] and the regulatory interactions among them analyzed by chromatin immunoprecipitation and gene expression profiling [30,31] or by a systematic screening using a collection of strains deleted for transcription factors [32]. The complexity of this transition is revealed by the involvement of chromatin reorganization via histone modifications [33,34,35].

Interestingly, Wor1 is involved in the **wo** transition and also in the adaptation to commensalism. This was suggested by the fact that the morphogenetic regulator Efg1 [36], also involved in the **wo** transition [37], is an important regulator of fungal loads in mouse gastrointestinal (GI) tract, as *efg1Δ* cells show higher fungal levels in the gut as compared with wild type cells [15]. *WOR1* overexpression (*WOR1^OE^*) from the *MET3* promoter provokes an en masse conversion of the white population to phloxine B positive cells in **a**/α mating type cells [29], thus, overriding repression by the **a**1/α2 dimer. *WOR1^OE^* a/α cells have an increased fitness in mouse GI tract [27], in striking contrast with the behavior of opaque cells derived from spontaneous switching of mating type homozygous cells that are rapidly lost [27]. Therefore, Wor1 has been suggested to mediate a morphological switch (termed GUT from gastrointestinally induced transition) via its increased expression upon passage through the mouse intestine, presumably converting a subset of *C. albicans* cells to the commensal status. GUT cells are morphologically slightly different from opaque cells and display a specific transcriptional program [27].

The changes occurring in *WOR1^OE^* cells that lead to an increase in fitness are not completely understood. Pande et al. reported significant changes in lipid, glucose, and iron metabolism genes and an increased expression of genes involved in N-acetyl glucosamine metabolism, a monosaccharide produced primarily by GI tract bacteria [27]. *WOR1^OE^* cells have an altered consumption of glucose and are sensitive to antimycin A [38], suggesting an altered balance in their respiratory metabolism. In this study, we characterized the phenotype of *WOR1^OE^* cells by different means. A proteomic analysis of *WOR1* overexpressing cells reveals changes in enzymes involved in the utilization of certain carbon sources and a repression of the glyoxylate cycle in these cells. We demonstrate that the growth of *WOR1^OE^* cells is altered in the presence of alternative carbon sources and show a role for the glyoxylate cycle in the production of opaque cells and the fitness of *C. albicans* cells in mouse GI tract.

## 2. Materials and Methods

### 2.1. Strains and Growth Conditions

All *C. albicans* strains used in this work are listed in Table 1. The construction of strains defective in *ICL1* or *MTLa1* or those overproducing Wor1 are described in the next section. In addition to the standard collection name, we provide a more descriptive name used in this manuscript.

Yeast strains were routinely grown at 37 °C in YPD liquid medium (2% glucose, 2% peptone, and 1% yeast extract). Growth was estimated by OD_600_ measurements. Drop tests for susceptibility/resistance or growth on carbon sources assays were performed by spotting 5 µL drops containing 10^5^ cells and ten-fold serial dilutions of stationary cells (grown for 18–20 h in YPD liquid medium at 37 °C) onto minimal medium (MM) agar plates (0.5% ammonium sulphate, 0.17% yeast nitrogen base without amino acids, and 2% agar) which was supplemented with 0.2% of sodium acetate, sodium citrate, ethanol, glycerol, glucose or 0.1% of olive oil/0.2% Tween 80. Plates were incubated at 37 °C for 72 h. Growth on xylose or trehalose was performed in 96-well microtiter plates by inoculating 10^3^ cells per well in MM supplemented with the indicated concentrations of xylose or trehalose and incubated at 37 °C for 2 or 6 days, respectively. Observation of white/opaque colonies was performed by spreading 200 colony forming units (CFUs) on YPD supplemented with 10 µg/mL of phloxine B (Sigma-Aldrich, city, state, country) and incubated for 48 h before photographs were taken. To induce white-opaque switching, 300 CFUs were spread per YPD plate (pH = 6) and supplemented with 5 µg/mL of phloxine B [43]. Plates were incubated at 28 °C for 48–72 h covered in aluminum foil. Fresh stools from mice collected during in vivo fitness assays were homogenized in sterile water and plated on SD medium (2% glucose, 0.5% ammonium sulphate, 0.17% yeast nitrogen base, amino acids, and 2% agar) supplemented with 20 µg/mL chloramphenicol.

### 2.2. Genetic Procedures

The pNIM1R-dTOM2 and pNIM1R-GFP [17] plasmids were both used to generate fluorescent *icl1Δ/Δ*-RFP and *icl1Δ/Δ*-pICL1-GFP labeled strains. These plasmids allow a repressible tetracycline dependent regulation of both fluorescent proteins (TET-OFF system) and carry the *SAT1* dominant marker. The products after digestion with *Kpn*I and *Ksp*I restriction enzymes were integrated at the *ADH1* locus of *icl1Δ/Δ* and *icl1Δ/Δ*-pICL1 *C. albicans* strains. Transformants were selected on YPD supplemented with 200 µg/mL of nourseothricin. Empty control vector pNRUe [40] and pNRUX-WOR1-myc [38] tetracycline repressible plasmids that carry the *URA3* marker were digested with *Kpn*I and *Ksp*I and products were integrated at the *ADH1* region of the MLC9 strain to generate *icl1Δ/Δ*-pNRUe and *icl1Δ/Δ*-WOR1^OE^ strains. Transformants were selected in SD plates with 2% glucose and without uracil. *C. albicans* transformation by electroporation was performed using described procedures [40].

Deletion of *MTLA1* gene was performed using the Transient CRISPR-Cas9 system [44,45]. The *CaCAS9* cassette was amplified from the plasmid pV1093 [46]. The sgRNA cassette expressed under the *SNR52* promoter and directed against the *MTLA1* region was constructed by single-joint PCR. A first round PCR was carried out by amplification of the SNR52 promoter and the 20 bp overlapping *MTL1* guide sequence by using SNR52/F forward primer and SNR52/R_MTL1 reverse primer. The second round PCR amplified the 20 bp complementary guide sequence and the sgRNA scaffold by using the sgRNA/F_MTL1 forward primer sgRNA/R reverse primer. First and second round PCRs were carried out by using the pV1093 plasmid as a template. Third round nested PCR for construction of the sgRNA expression cassette used both PCR products and SNR52/N forward primer and sgRNA/N reverse primer. The repair template cassette which contains the *SAT1* selection marker and 80 bp *MTLA1* additions on both sides was amplified from pNIM1R-RFP by using MATa1_del_F forward primer and MATa1_del_R reverse primer. The guide sequence from the sgRNA cassette hybridizes at the *MTLA1* region enabling CaCas9 to break the double strand, triggering the homology-directed repair by integration of the *SAT1* cassette. PCR products of CaCAS9, sgRNA, and *SAT1* repair template cassettes were used to co-transform *icl1Δ/Δ* and *icl1Δ/Δ*-pICL1 strains following the same procedure and selected on YPD supplemented with nourseothricin; *icl1Δ/Δ*-pICL1 is a reintegrant strain in the *RPS10* locus where *ICL1* expression is driven from its own promoter. Genomic DNA from transformants was used to determine *MTLA1* deletion by PCR using oRS108 forward primer (inside *MTLA1* sequence), Comp_del_F forward primer (inside *SAT1* sequence), and oRS109 reverse primer (outside of recombination site). All primers are listed in Table 2.

### 2.3. Protein Extraction and Proteomics Analysis

Proteins were extracted from 300 µL overnight cultures (18–20 h of growth in YPD medium at 37 °C). These conditions were chosen for proteomic analysis as they gave a superior reproducibility in test pilot studies. Under these conditions, most of the cells remained in yeast form. Cell extracts were obtained by using glass beads in a Fast prep breaker, as described previously [47]. The supernatant was collected, and the protein concentration was measured using a Bradford assay. Protein extracts were precipitated with MeOH/chloroform and resuspended in 8 M Urea. Protein extracts (100 µg) were reduced with 10 mM DTT (Sigma-Aldrich) for 1 h at 37 °C followed by alkylation with 55 mM iodoacetamide (Sigma-Aldrich) for 1 h in the dark at room temperature. Proteins were digested with 1/50 (*w*/*w*) of recombinant trypsin (Roche Molecular Biochemicals, Mannheim, Germany) in 25 mM ammonium bicarbonate adjusted to pH = 8.5 and incubated overnight at 37 °C. Digested peptides were desalted and concentrated, as described before [48]. Peptides were analyzed by reverse phase liquid chromatography electrospray ionization tandem mass spectrometry (RP-LC-ESI-MS/MS) on a nano Easy-nLC 1000 (ThermoScientific, San Jose, CA, USA,) coupled to a Q-Exactive HF mass spectrometer (Thermo Scientific). Desalted peptides were concentrated by loading them on an Acclaim PepMap 100 column (Thermo Scientific, 20 mm × 75 μm inner diameter, 3 µm diameter C18 and 100 Å pore size). Peptides were separated and eluted on a C18 Picofrit column (Thermo Scientific Easy Spray Column, PepMap RSLC C18 500 mm × 75 μM inner diameter, 2 μM diameter, 100 Å pore size) with an integrated spray tip at a flow rate of 250 nL/min for 240 min. Buffer A (2% acetonitrile and 0.1% formic acid) and buffer B (0.1% formic acid on acetonitrile, gradient from 2 to 40%) were used. Peptides were detected with a Q-Exactive mass spectrometer at a *m/z* range of 350–2000 Da with a mass resolution of 60,000 and acquired using data-dependent acquisition (DDA). The 15 most abundant precursors with charges of 2–6+ (threshold 8 × 10^3^) were selected for higher energy collisional dissociation (HCD) fragmentation with a dynamic exclusion of 27 s. The normalized collision energy was 27%.

Peptide identification was carried out by using the Mascot v. 2.6.1 search engine (MatrixScience) through the Protein Discoverer 2.2 Software (Thermo Scientific) and the CGD21 database from http://www.candidagenome.org accessed on date 10 April 2018. The following parameters were used: tolerances of 10 ppm for precursor ions and 0.02 Da for MS/MS fragment ions, up to two missed cleavage sites from trypsin digestion and allowing optional methionine oxidation and fixed carbamidomethylation of cysteine. The acceptance criteria for protein identification were an FDR < 1% and at least one unique peptide identified with high confidence (percolator *q*-value < 0.01). Protein quantification was carried out, as previously described [48]. Protein extracts were treated for mass spectrometry at the Proteomics Unit of the Universidad Complutense de Madrid.

CGD was the main database used for the functional classification. GO Slim Mapper tool was used for the analysis by cellular component, while the ontological enrichment analysis by biological process was carried out by PathoYeastract, Rank by GO.

### 2.4. In Vivo Procedures

In vivo fitness assays were performed on 7–10-week-old female mice C57BL/6 (Charles River Laboratories España S.A.U, St. Cugat del Vallés, Barcelona, España) [17]. In these experiments, two different genetically labeled strains (either GFP or RFP) were administered by gavage and the relative colonization of each strain was determined by counting green/red colonies, as described by [17]. Genetic labeling was stable and resulted in a rather homogenous fluorescence in all cells leading to easily distinguishable colonies on solid medium. Colonization assays were started four days before with antibiotic pretreatment (2 mg/mL streptomycin, 1 mg/mL bacitracin, and 0.1 mg/mL gentamycin) and 0.25 mg/mL fluconazole added to drinking water. One day before inoculation, fluconazole was retired and a single gavage of 10^7^ cells in 100 µL PBS was then intragastrically inoculated. Fresh stool samples were collected from each mouse every 2–4 days and mechanically homogenized in PBS. Ten-fold serial dilutions were plated on SD medium supplemented with 20 µg/mL chloramphenicol and incubated at 37 °C for 2 days. The fungal population was quantified by CFU determination. Experiments involving animals were carried out in the animal facility at the Medical School of the Universidad Complutense de Madrid in strict accordance with the regulations “Real Decreto 1201/2005, BOE 252” for the Care and Use of Laboratory Animals of the “Ministerio de la Presidencia,” Spain. The commensalism model used in these experiments was approved by the Animal Experimentation Committee of the University Complutense of Madrid (CEA 33-2015) and Comunidad de Madrid according to Artículo 34 del RD 53/2013 (PROEX 226/15). The treatments did not result in disease and procedures minimized any suffering. The number of animals used in every experiment was adjusted to a minimum for ethical reasons.

## 3. Results

### 3.1. Analysis of Proteome in WOR1 Overexpressing Cells

**Figure 1:**
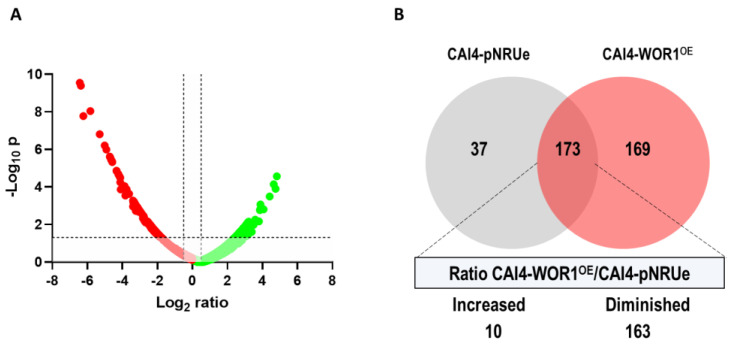
Proteome changes in cells overproducing Wor1. (**A**) Volcano plot of differentially abundant proteins (CAI4-*WOR1^OE^*/CAI4-pNRUe ratio). The log_10_ of the *p*-value is plotted against abundance (log_2_ ratio) for each individual protein. Proteins that were found in lower abundance in *WOR1^OE^* cells are shown in red whereas proteins with higher abundance are shown in green. Horizontal dotted line indicates the limit for *p*-values (*p* = 0.05, that correspond to - log_10_ p = 1.3). Vertical dotted lines indicate the log_2_ ratio = ±0.5 threshold. (**B**) Venn diagram with the number of proteins identified exclusively in one strain or in both strains, based on a variability ratio <30% between replicates and a Mascot score > 13.

To characterize the phenotype of *WOR1^OE^* cells, we used strain CAI4-WOR1^OE^ [38], a CAI4-derived strain (being therefore mating type **a**/α cells) where the expression of an ectopically integrated wild type allele of *WOR1* is under the strong and tightly regulated TET^OFF^ promoter, a tetracycline repressible version of the TET promoter [49]. We performed a proteomic analysis of these cells after growing for 18–20 h in YPD medium at 37 °C as compared with the same strain carrying the empty vector (CAI4-pNRUe). Whole cell extracts were obtained from these cells and subjected to LC-MS/MS (liquid chromatography mass spectrometry analysis, Thermo Scientific, San Jose, CA, USA). Among the 3254 identified proteins, only 3190 could be quantified. To determine the number of proteins that decreased or increased in the CAI4-WOR1^OE^ strain, we used the log_2_ ratio < −0.5 or log_2_ ratio > 0.5, respectively, and only considered peaks with a CAI4-WOR1^OE^/CAI4-pNRUe (control strain) abundance ratio with a statistical significance *p* < 0.05 (expressed as −log10 *p* > 1.3). This resulted in 242 proteins that are represented in the Volcano plot (Figure 1A). We also considered proteins that showed a variability ratio < 30% between replicates, had more than one identified peptide, and a Mascot score >13 resulting in 379 proteins; 37 were only detected in the CAI4-pNRUe control strain (abundance ratio expressed in log2 < −6.64), 169 were only detected in the WOR1^OE^ strain (log2 > 6.64), and 173 were common to both. Within this subset, 163 proteins were less abundant in the CAI4-WOR1^OE^ strain, while only a few (10) were increased (Figure 1B).

Among those proteins less abundant in *WOR1^OE^*, we identified cytoplasmic (74), nuclear (32), mitochondrial (25) or ribosomal proteins (24), as well as proteins located in the mitochondrial envelope (15), plasma membrane (14), cell wall (13), cytoskeleton (7), vacuole (3), peroxisome (2), endoplasmic reticulum (2), or Golgi (2). Among the 10 proteins identified with highest abundance in the CAI4-WOR1^OE^ strain, we identified proteins located in the nucleus (5), cytoplasm (3), and mitochondria (2). Wh11, a protein only found in white budding-phase cells and absent in opaque budding phase cells, or Adh5, regulated by white-opaque switch, were less abundant in cells overproducing Wor1, thus, validating our data.

Our analysis revealed a significant decrease in the abundance of proteins involved in carbon metabolism and nutrient acquisition processes, such as Icl1 (isocitrate lyase), Pck1 (phosphoenolpyruvate kinase), Adh5 (alcohol dehydrogenase), Glx3 (glutathione-independent glyoxalase), and the high affinity transporters for glucose Hgt1 and Hgt19. We also found proteins within this subset related to mitochondria at different steps such as the import and sorting of proteins of nuclear origin. We also found within this subset the translocase of the outer membrane (TOM) complex that mediates translocation of proteins across or into the outer membrane (OM). In particular, Tom22 or the orf19.6062; orf19.6062, the putative Tim23 translocase subunit which takes in proteins with a cleavable mitochondrial targeting sequence (MTS) directing them into the matrix or the inner membrane. We also found Tim9, Tim13, and Tim22 together with small soluble proteins in the IMS (called Tim), which deliver into the IM the proteins of the so-called mitochondrial carrier family (MCF) that lacks a cleavable MTS. Others were enzymatic systems involved in mitochondrial oxidative phosphorylation (OXPHOS), such as the cytochrome-c oxidase Cox4, or Cyb5 (cytochrome b5) that form part of the electron chain, or C1_06840cp_a, C2_01720cp_a, and C7_01610wp_that are involved in the assembly of complex IV cytochrome-b oxidase, or Atp20 (subunit g of the mitochondrial membrane ATP synthase). We also found some proteins that form part of mitochondrial ribosomes such as C1_0670wp_a y Cr_04580wp_a. A set of proteins related to the cellular response to stress were also decreased, such as the oxido reductases Pst2 and Cip1, the catalase Cat1, and the thioredoxin peroxidase Dot5.

A total of 169 proteins were only found in the *WOR1^OE^* strain. The analysis by cellular component determined that most of the proteins were located in the nucleus (60) followed by the cytoplasm (57), membrane (37), chromosome (22), nucleolus (13) plasma membrane (12), Golgi apparatus (9), mitochondria (8), endoplasmic reticulum (8), cytoskeleton (8), vacuole (5), and peroxisome (3), among others (Supplementary List).

### 3.2. WOR1 Is Involved in the Use of Non-Fermentable Two Carbon Sources 

**Figure 2:**
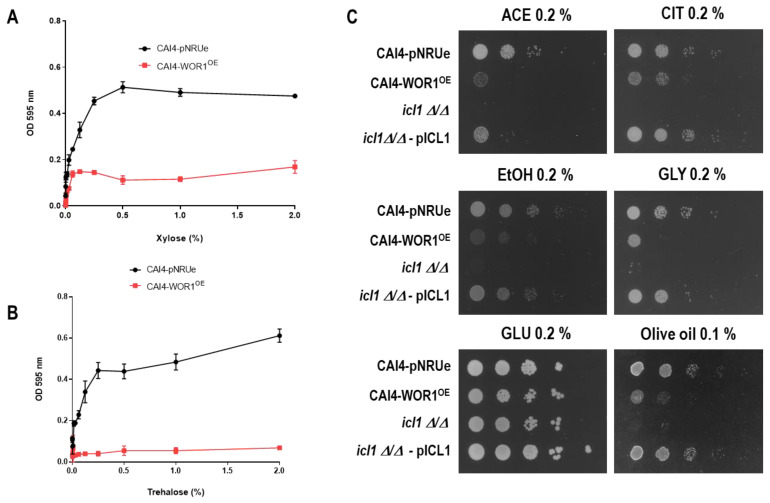
Comparative growth of strains in different carbon sources. Growth of pNRUe and *WOR1^OE^* strains on MM medium supplemented with different concentrations of (**A**) xylose or (**B**) trehalose. The 96-well microtiter plates were incubated for 2 days (xylose) or 6 days (trehalose). Values are mean ± standard deviation of three independent replicates. (**C**) 10^5^ cells and ten-fold dilutions from overnight growing cells from CAI4-pNRUe, CAI4-*WOR1^OE^*, *icl1****Δ/Δ,*** and *icl1****Δ/Δ***-pICL1 strains were spotted onto MM plates without amino acids and supplemented with 0.2% of sodium acetate (Ace), sodium citrate (CIT), ethanol (EtOH), glycerol (GLY), glucose (GLU), or olive oil. Plates were incubated at 37 °C for 72 h before being scanned.

As proteomics revealed changes in the enzymes involved in carbon metabolism, we tried to determine the behavior of cells in specific carbon sources. We determined the ability to grow on minimal media at 37 °C using trehalose and xylose as carbon sources (Figure 2A,B) by measuring O.D. The O.D. reached under the conditions tested (2 days for xylose and 6 days for trehalose) by CAI4-WOR1^OE^ strain grown in xylose containing medium was 30–40% of the parental strain; these defects were more evident in the presence of trehalose, as *WOR1^OE^* cells were completely unable to use this disaccharide as a carbon source (Figure 2B).

Interestingly, our proteome analysis also revealed that the isocitrate lyase Icl1, a key enzyme in the glyoxylate cycle, showed reduced levels when *WOR1* was overexpressed (the log2 ratio *WOR1^OE^*/pNRUe was −3.76). The glyoxylate cycle allows cells to use two carbon sources bypassing two sequential decarboxylation steps in the Krebs cycle [41]. Therefore, we analyzed the growth of *WOR1^OE^* cells on different carbon sources in solid media. The overexpression of *WOR1* resulted in a significant growth defect in nonfermentable carbon sources such as acetate, citrate, ethanol, and glycerol in a standard drop assay (Figure 2C), a phenotype resembling those of *icl1Δ/Δ* mutants [41] (Figure 2C). Such differences were not observed in the presence of the fermentable carbon source glucose. Growth defects were also observed in olive oil supplemented minimal medium, consistent with the role of the glyoxylate cycle in metabolizing acetyl-CoA intermediates derived from the β-oxidation of fatty acids. We conclude that overexpression of *WOR1* in *C. albicans* alters the carbon metabolism of the cells by reducing its ability to use some carbohydrates and, mainly, two carbon sources probably via a reduction in the glyoxylate shunt.

### 3.3. icl1Δ/Δ mutants Fail to Produce Phloxine B+ Cells in WOR1 Overexpressing Cells

**Figure 3:**
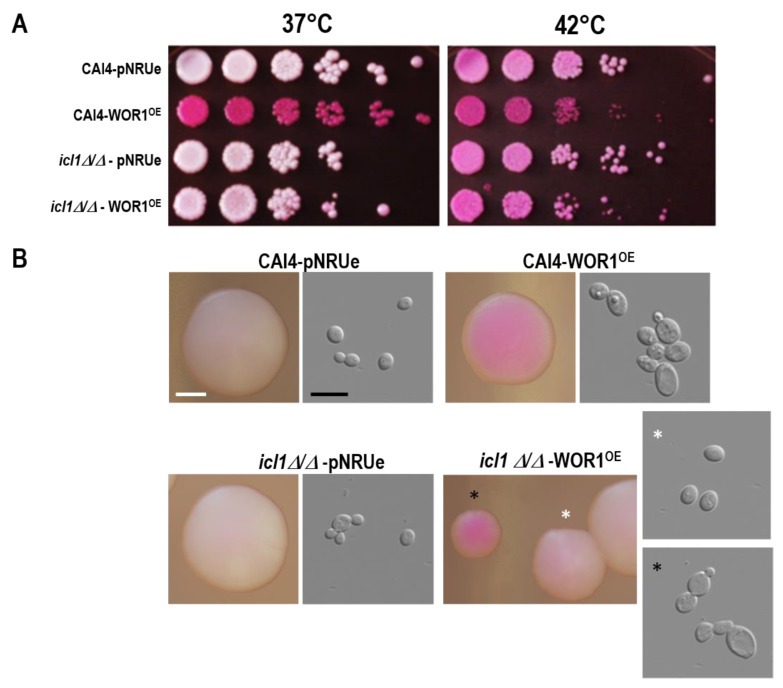
Effect of *WOR1* overexpression on phloxine B+ cell formation in wt and *icl1Δ/Δ* backgrounds. (**A**) Production of phloxine B + cells. Cells were grown for 18–20 h at 37 °C. Cells of CAI4-pNRUe, CAI4-WOR1^OE^, *icl1Δ/Δ,* and *icl1Δ/Δ*-WOR1^OE^ strains were spotted onto YPD supplemented with 10 µg/mL of phloxine B and incubated at 37 °C for 48 h. (**B**) 200 CFUs were spread on the same media and incubated at 37 °C for 48 h. White scale bar stands for 0.5 mm; black scale bar stands for 10 µm. Photographs of individual colonies and their corresponding cells are shown and marked with either a white or black asterisk.

Given the defects in the utilization of carbon sources by *WOR1* overexpressing cells, we analyzed the role of the glyoxylate cycle in *WOR1^OE^*. For this purpose, we ectopically expressed *WOR1* from the constitutive TET^OFF^ promoter in *icl1Δ/Δ* mutants and noticed that the production of phloxine B positive cells in *icl1Δ/Δ*-*WOR1^OE^* cells was defective at 37 °C, with most of the cells being mainly negative (or very slightly pink), indicating a defect in retaining this fluorochrome (Figure 3A). This effect was observed both at 37 °C and 30 °C (not shown) and was largely suppressed by high temperature (42 °C). Cells from the *icl1Δ/Δ*-*WOR1^OE^* mutant at 37 °C showed a heterogeneous colony size, with a reduced number of small colonies that retained more actively the fluorochrome with cells resembling those found in *WOR1^OE^* strains (with larger cells with a larger vacuole, but not true elongated as opaque cells). However, the most abundant bigger colonies were white and phloxine B negative with typical rounded cells (Figure 3B); however, this did not occur with wild type *WOR1^OE^* cells being all of them phloxine B+.

**Figure 4:**
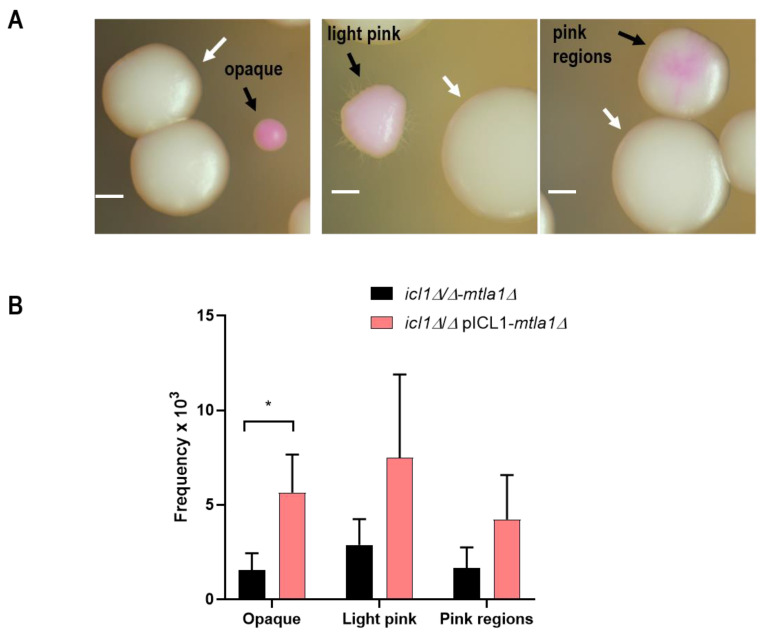
Induction of white-opaque conversion in *icl1Δ/Δ-mtla1* and *icl1Δ/Δ*/*ICL1-mtla1* strains. 300 CFUs were spread onto YPD pH = 6 plates supplemented with 5 µg/mL phloxine B and incubated in the dark for 2 days at 28 °C and 5% CO_2_. The upper picture (**A**) indicates the appearance of the different counted colonies as standard white colonies (white arrows) or pink colored colonies (black arrows), either opaque, light pink, or mixed colored colonies (pink regions) in *icl1Δ/Δ-mtla1*. White scale bar stands for 1 mm. Data (**B**) are shown as mean with standard deviation (SD) from six independent experiments (*n* ≈ 2500 colonies from each strain per experiment). The frequency (colonies of the indicated type/1000 total colonies) is represented. A *t*-test was used to determine the significance (*p* = 0.021, * *p* < 0.05).

These results, obtained from an artificial and ectopically expressed *WOR1*, prompted us to investigate whether the glyoxylate cycle could be involved in opaque cell formation. As this spontaneous conversion occurs in a/α hemizygous mating-type cells, we deleted the mating gene *MTLa1* in *icl1Δ/Δ* mutants and *icl1Δ/Δ*/*ICL1* reintegrants (see Material and Methods) and analyzed the production of opaque cells at 28 °C in 5% CO_2_ atmosphere. As shown in Figure 4, *icl1Δ/Δ-mtla1* mutants switched 4.4 +/− 2.1 times less frequently than *icl1Δ/Δ*/*ICL1-mtla1* heterozygous cells. No statistical differences were found with light pink colonies. In addition, opaque cells appeared as smaller colonies as compared with white cells, suggesting the importance of a functional glyoxylate cycle in opaque cells. Collectively, these results indicate that the conversion to the opaque phase is influenced by a functional glyoxylate cycle and suggests that *WOR1^OE^* regulates this conversion, at least in part, via repression of this pathway.

### 3.4. icl1Δ/Δ Mutants Are Defective in Gastrointestical Colonization

**Figure 5:**
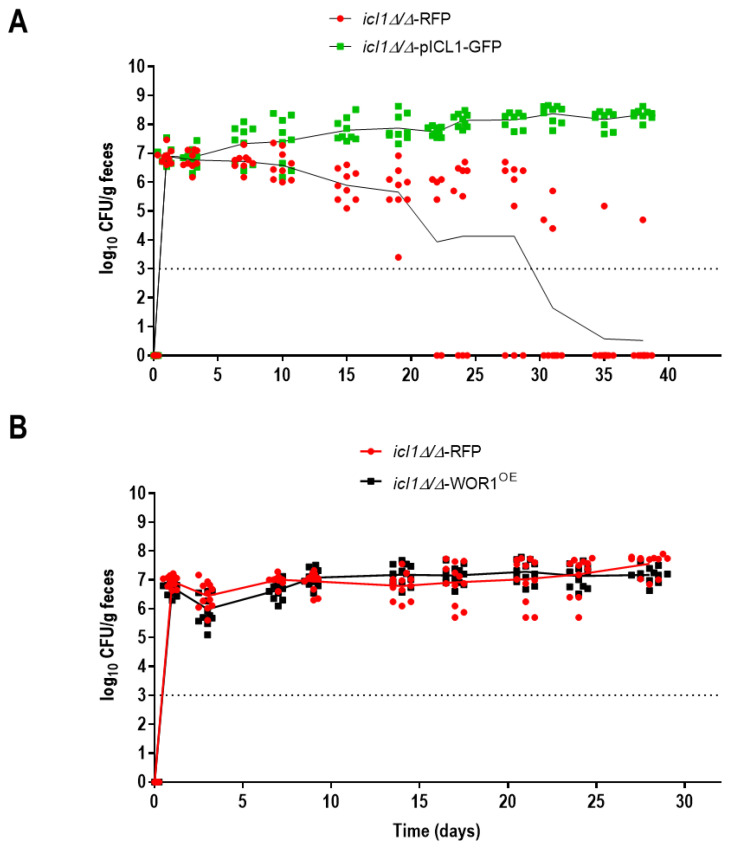
Gastrointestinal colonization of *icl1Δ/Δ* mutants. Oral antibiotic therapy was given to mice starting 4 days before inoculation by gavage of 10^7^ cells of a 1:1 mixture of *icl1Δ/Δ*-RFP and *icl1Δ/Δ*-pICL1-GFP (*n* = 9) (**A**) or *icl1Δ/Δ*-RFP and *icl1Δ/Δ*-WOR1^OE^ (*n* = 12) (**B**). The dotted line indicates the limit of detection of the method used.

As *WOR1^OE^* has been shown to influence gastrointestinal colonization improving the fitness of *C. albicans* (deletion of *WOR1* reduces fitness) [27], we wondered whether deletion of *ICL1* would also affect colonization. For this purpose, we used a competitive fitness assay between *icl1Δ/Δ* and *icl1Δ/Δ*-p*ICL1* mutants. Cells were genetically labeled with GFP or RFP and inoculated as a 1:1 inoculum of both cell types by gavage to mice. As shown in Figure 5A, the *icl1Δ/Δ* mutant showed fitness defects as compared with isogenic *icl1Δ/Δ*-p*ICL1* cells, with a continuous and slow reduction in fungal colonization (determined by CFUs in stools). The *icl1Δ/Δ* cells were outcompeted by the reintegrated *ICL1* strain, being evident from day 15 onwards until they were not detected in most mice after ≈30 days of growth. To determine whether *icl1Δ/Δ* colonization defects could be restored by *WOR1^OE^*, we did a similar experiment with *icl1Δ/Δ* and *icl1Δ/Δ*-*WOR1^OE^* cells. As shown in Figure 5B, the overproduction of *WOR1* does not outcompete an *icl1Δ/Δ* mutant. 

These results demonstrate a relevant role of Icl1 in facilitating commensalism of *C. albicans* in the gastrointestinal tract of mice.

## 4. Discussion

The ability to colonize the mammalian gastrointestinal tract is a key trait in *C. albicans*, as many of the nosocomial systemic fungemia have an endogenous origin via dissemination from the gastrointestinal pool. Therefore, understanding which factors promote the colonization of this fungus in the gut may have important practical consequences.

The Wor1 regulator has been described as a factor promoting increased fitness in mouse intestine [27] and it triggers in vivo an epigenetic switch that enables colonization. Our in vitro proteomic analyses revealed that overexpression of *WOR1* is associated with a change in the pattern of carbon source assimilation, with a reduction in the ability to use certain fermentable and 2C sources. These changes are in concordance with those already observed in a previous transcriptomic analysis [27], with a downregulation of genes involved in the catabolism of glucose and also with some changes observed in an extensive opaque cell phenotypic profiling study [50]. Our proteomic analysis revealed a significant decrease in proteins involved in carbon metabolism and nutrient acquisition processes. In particular, we found Icl1 (isocitrate lyase), Pck1 (phosphoenolpyruvate kinase), Adh5 (alcohol dehydrogenase), Glx3 (glutathione-independent glyoxalase), or the high affinity transporters for glucose Hgt1 and Hgt19. Pck1 and Glx3 participate in the metabolism of pyruvate, the final product of glycolysis. Pyruvate can enter the mitochondria and produces acetyl-CoA or remains in the cytoplasm to generate lactate or ethanol involving Adh5 activity. The differential metabolic profile observed in the *WOR1*^OE^ strain suggests a decrease in glycolysis and an increase in gluconeogenesis, in accordance with the transcriptomic data reported by Pande and co-workers [27]. Moreover, the decrease in glycolytic enzymes may explain the hypersensitivity to sodium azide exhibited by *WOR1^OE^* cells [38]. These cells, thus, become more dependent on the ATP generated by the electron transport chain.

Icl1 is a key enzyme in the glyoxylate pathway which enables the use of 2C sources bypassing the decarboxylation steps of the Krebs cycle enabling gluconeogenesis. It is therefore important for growth in nutrient-limited environments such as those that occur inside phagocytic cells where lipids and amino acids can be used as an alternative to hexose depletion and the glyoxylate cycle, β-oxidation, and gluconeogenesis metabolic pathways are required to use less favored carbon sources. These pathways have been shown to be important during systemic infection since deletion of genes encoding key enzymes in the pathways, such as *FOX2* (β-oxidation), *ICL1* (glyoxylate cycle), or *FBP1* (gluconeogenesis), confer virulence defects to a different extent [42]. Thus, it seems that *C. albicans* acquires and assimilates nonfermentable (non-sugar) compounds not only as an alternative carbon source during infection [41] but also during colonization, as shown by the reduced fitness of *icl1Δ/Δ* mutants in mouse GI. The glyoxylate cycle takes place in peroxisomes which are involved in fatty acid metabolism [51], and therefore one explanation for this result would invoke fatty acid assimilation. The availability of glucose is scarce in rodent food and would be very limited in distal regions of the GI where the use of alternative carbon sources (such as fatty acids) could be relevant. The concentration of fatty acids changes along the GI tract and it has been shown that a coconut oil diet enriched in medium-chain fatty acids alters the fungal load of *C. albicans* in mouse GI [52]. The ability to use 2C compounds or other carbon sources such as glycerol could be, additionally, important only in specific locations of the gut (e.g., distal part) or after a specific period of adaptation of the fungal cells [53]. Since *C. albicans* usually depends on the mitochondrial oxidative phosphorylation to obtain energy, metabolic adaptation is crucial for *C. albicans* to survive and colonize the intestine. We found that *WOR1* overexpression caused a reduction in the amount of Icl1. However, as deletion of *ICL1* impairs the use of nonfermentable carbon sources this result is in apparent contradiction with the proposed role of *WOR1* in promoting colonization. This would indicate the existence of *ICL1*-independent but *WOR1*-dependent mechanisms for adaptation to the GI tract, that is, downregulation of the glyoxylate cycle would be compensated by other mechanisms that remain to be discovered. Such mechanisms could involve adhesion to the mucosal surfaces, resistance to stressful conditions found in the GI tract (detergents such as bile salts, oxidants, pH, oxygen availability, etc.) or competition with the endogenous microbiota. In any case, caution must be taken while analyzing these data, as our proteomic analysis was carried out under well-defined laboratory conditions (as occurs with related studies on opaque and GUT cells) and the actual conditions in the mammalian gut are clearly different and complex.

The proteomic changes observed were only partially coincident with those observed for previous transcriptional studies on opaque cells [21,22,23] and GUT cells [27]. A detailed analysis of coincidences and discrepancies is shown in the Supplementary List. It should be noted that, *ICL1* is described as an opaque specific gene in two of these publications [21,23] and its expression is augmented in GUT cells versus white a/α cells but not versus opaque cells [27], suggesting it could be related to *WOR1*. The expression of several white specific genes is decreased in *WOR1^OE^* in our proteomic analysis (*ASR1*, *HSP21*, *IFE2*, *HSP12*, and *GIS2*) but there are also some opaque genes with reduced levels such as the mentioned *ICL1*, *PST2* (encoding a flavoredoxin), *FDH1* (encoding a formate reductase), and orf19.94 (unknown function). Regarding those proteins with increased abundance in this study in *WOR1^OE^*, most of them are identified as opaque specific (e.g., *OP4*, a usual reporter of the opaque phase) but there are some white specific genes such as orf19.1691 (a putative plasma membrane protein) or *TPO4* (a putative spermidine transporter). This analysis reveals an overall reasonable degree of concordance among these studies but also highlights discrepancies as expected from different morphogenetic programs. In addition, both the methodology (mRNA, protein) and the experimental conditions used for the study (temperature, nutrient, and phase of growth) can clearly influence the outcome and interpretation of the results.

Another important result from this work is that the glyoxylate cycle participates in the generation of phloxine B positive cells, which is a characteristic of *WOR1* overexpression. The ability to retain this fluorochrome is also a landmark of opaque cells; however, opaque cells differ from *WOR1^OE^* cells by their elongated morphology, heat sensitivity, the existence of protuberances in the surface, and mating competent status; they are able to form mating projections and are generated via a spontaneous epigenetic switch through mating type repression release and not via ectopic overexpression of *WOR1*. They have a characteristic transcriptional program [21,22] which is only partially overlapping with those of GUT cells [27]. As deletion of *ICL1* in hemizygous α-mating-type cells reduces the frequency of opaque cell formation and overexpression of *WOR1* partially restores the generation of phloxine B positive cells, these results clearly point to a positive role of Icl1 in the **wo** transition. Nevertheless, when the *ICL1* gene was overexpressed in a *wor1* mutant, cells did not become phloxine B positive (data not shown), indicating the dominant effect of *WOR1* over *ICL1* in **wo** switching. As the glyoxylate cycle takes place in peroxisomes, organelles involved in lipid beta-oxidation among other functions [54], a proposed explanation is that *C. albicans icl1Δ/Δ* mutants are somehow altered in lipid homeostasis which would render cells less permeable to phloxine B. In any case, as *WOR1^OE^* does not restore colonization levels of *icl1Δ/Δ* mutants, this indicates that phoxine B positiveness cannot be taken as a trait of the adapted GI form in *C. albicans* per se. 

In conclusion, we show that the glyoxylate shunt pathway is involved in colonization of mouse GI by mechanisms that are independent of the transcriptional **wo** regulator Wor1 and the generation of phloxine B positive cells. Understanding how these changes promote adaptation to the gastrointestinal niche open the possibility to control *C. albicans* colonization for therapeutic purposes.

## Figures and Tables

**Table 1:** Strains of *C. albicans* used in this study.

Strain Name	Common Name in Manuscript	Background Strain and Genotype	Source
CAI4		*ura3Δ::imm434/ura3Δ::imm434*	[39]
CAI4-pNRUe		[CAI4] *ADH1/adh1::tTATET^PR^-myc-URA3*	[40]
CAI4-RFP		[CAI4] *ADH1/adh1:: tTATET^PR^-dTOM2-URA3*	[38]
CAI4-WOR1^OE^		[CAI4] *ADH1/adh1:: tTATET^PR^-WOR1-myc-URA3*	[38]
MLC9		[CAI4] *icl1::hisG/icl1::hisG*	[41]
MRC10	*icl1Δ/Δ*	[CAI4] *icl1::hisG/icl1::hisG RPS10/rps10::URA3*	[42]
MRC11	*icl1Δ/Δ*-pICL1	[*icl1Δ/Δ*] *RPS10/rps10::ICL1-URA3*	[42]
SHV1	*icl1Δ/Δ*-pICL1-GFP	[*icl1Δ/Δ*-pICL1] *ADH1/adh1:: tTATET^PR^--GFP-myc-SAT1*	This study
SHV2	*icl1Δ/Δ*-RFP	[*icl1Δ/Δ*] *ADH1/adh1:: tTATET^PR^-dTOM2-SAT1*	This study
SHV3	*icl1Δ/Δ*-pNRUe	[*icl1Δ/Δ*] *ADH1/adh1::tTATET^PR^-myc-URA3*	This study
SHV4	*icl1Δ/Δ*-*WOR1^OE^*	[*icl1Δ/Δ*] *ADH1/adh1:: tTATET^PR^-WOR1-myc-URA3*	This study
SHV5	*icl1Δ/Δ-mtla1Δ*	[*icl1Δ/Δ*] *mtla1∆::SAT1*	This study
SHV6	*icl1Δ/Δ*-pICL1-*mtla1Δ*	[*icl1Δ/Δ*-pICL1] *mtla1∆::SAT1*	This study

**Table 2:** Primers used in this study.

Primer Name	Sequence (5′→3′)
SNR52/F	AAGAAAGAAAGAAAACCAGGAGTGAA
SNR52/R_MTL1	CTCACGCTTCAATTGTAAGACAAATTAAAAATAGTTTACGCAAGTC
sgRNA/F_MTL1	TCTTACAATTGAAGCGTGAGGTTTTAGAGCTAGAAATAGCAAGTTAAA
sgRNA/R	ACAAATATTTAAACTCGGGACCTGG
SNR52/N	GCGGCCGCAAGTGATTAGACT
sgRNA/N	GCAGCTCAGTGATTAAGAGTAAAGATGG
MATa1_del_F	TTTCTGCGTATTGTGATAAATAACTTTTTTTCCCTCTAAAAATATTGATTAGAGGCACAAAATAAAAATCACCTTCAACCCGTCAAAACTAGAGAATAATAAAGAAAACG
MATa1_del_R	CCCACCAAGACATGTTACGAATAGATCTATTAGTTAGCAATATTCTGTTTGATAATACATACCCAAACTCTTATTTGGGAGCAGGACCACCTTTGATTGTAAATAG
Comp_del_F	CACGTATAAAACTAGACCTCAAGTCTCG
oRS108	ATGAACTCAGAAATAGAAAGTAGC
oRS109	CTAGGTTGAATTTGAACTTGATTT

## Data Availability

Not applicable.

## Supplementary Materials

The following are available online at https://www.mdpi.com/article/10.3390/jof7070502/s1, Supplementary List.
