# Supplementary material for: The Glyoxylate Cycle Is Involved in White-Opaque Switching in Candida albicans
Source: J Fungi (Basel). 2021 Jun 24;7(7):502. doi: 10.3390/jof7070502 (PMC8304919; doi:10.3390/jof7070502)
Supplement: Supplementary file 1 [file jof-07-00502-s001.zip › jof-1273019-supplementary.pdf]

Supplementary List

379 identified proteins with a p-value <0.05. Abundance ratio is expressed in log2 and p-value ratio is expressed in -log10 with a coefficient of variance <30% between replicates, more than one identified peptide and Score Mascot > 13 were considered

			Reported Specificity		(Wh v. Op)	Relative Expression Pande et al.[#]				
**Systematic Name**	**Standard Name**	**Assembly 19/21 Identifier**	**Lan et al.[*]**	**Tsong et al.[**]**	**Tuch et al.[***]**	***WOR1*- OE *MTL* a/α GUT/ WT*MTL* a/α Wh**	***WOR1*- OE *MTL* a/α GUT/ *MTL* a Op**	**Abundance Ratio WOR1^{OE}/pNRUe (log2)**	**Ratio WOR1^{OE}/pNRUe p-value (-log10)**	**Description**
**Only in CAI4 pNRUe**										
C1_06070W_A		orf19.2438				1.63	1.57	-6.64	17	Uncharacterized ORF; Protein of unknown function
C3_02760C_A		orf19.267				0.99	1	-6.64	17	Protein required for normal filamentous growth
CR_04020C_A		orf19.485				0.94	0.98	-6.64	17	Uncharacterized ORF; Protein of unknown function
C3_04080W_A		orf19.913.2			Wh	0.29	0.64	-6.64	17	Uncharacterized ORF; Ortholog of subunit 6 of the ubiquinol cytochrome-c reductase complex, a component of the mitochondrial inner membrane electron transport chain; Hap43-repressed gene
C5_03870C_A		orf19.1107			Wh	1.06	1.23	-6.64	17	Uncharacterized ORF; Protein of unknown function; Spider biofilm induced
C4_04050C_A	RHD3	orf19.5305	Op	Op	Op	6.03	0.67	-6.64	17	GPI-anchored yeast-associated cell wall protein; induced in high iron; clade-associated gene expression; not essential for cell wall integrity; fluconazole-repressed; flow model and Spider biofilm repressed
C2_05480C_A		orf19.3577.1				1.2	9.92	-6.64	17	Uncharacterized ORF; Protein of unknown function
C1_09790C_A		orf19.4844				1.22	1.77	-6.64	17	Uncharacterized ORF; Protein of unknown function
C1_02890C_A		orf19.2978				1.08	1.29	-6.64	17	Uncharacterized ORF; Protein of unknown function; Hap43-repressed gene
C2_01680C_A		orf19.1479				0.73	0.8	-6.64	17	Uncharacterized ORF; Ortholog of the mitochondria localized S. cerevisiae Pib2 protein of unknown function; has a FYVE zinc finger domain; Spider biofilm induced
CR_04730W_A		orf19.6318				0.99	0.83	-6.64	17	Uncharacterized ORF; Protein of unknown function
C6_03240W_A		orf19.5611				1.07	1.09	-6.64	17	Uncharacterized ORF; Predicted 3-methylbutanol:NAD(P) oxidoreductase and methylglyoxal reductase (NADPH-dependent); role in ergosterol metabolic process; early stage flow model biofilm induced; Spider biofilm induced
C2_08850C_A		orf19.225				0.81	0.91	-6.64	17	Uncharacterized ORF; Predicted 2-hydroxyacid dehydrogenase; Hap43-repressed gene
C3_02290W_A		orf19.1617				1.05	0.74	-6.64	17	Protein similar to S. cerevisiae Ydr282cp; transposon mutation affects filamentous growth; Hap43p-repressed gene
C3_04330C_A		orf19.5876				1.56	1.08	-6.64	17	Uncharacterized ORF; Protein of unknown function; Cyr1-repressed; induced by alpha pheromone in SpiderM medium; rat catheter and Spider biofilm induced
C1_11320C_A		orf19.670.2				0.83	0.52	-6.64	17	Uncharacterized ORF; Protein of unknown function; hypoxia, Hap43-repressed; ketoconazole induced; induced in oralpharyngeal candidasis; 16h flow model biofilm repressed, late-stage flow model biofilm induced; rat catheter and Spider biofilm induced
C1_01280C_A	MED22	orf19.3317				1.26	1.3	-6.64	17	Uncharacterized ORF; RNA polymerase II mediator complex subunit
CR_06780W_A		orf19.1856				1.03	1.06	-6.64	17	Uncharacterized ORF; Protein of unknown function
C1_10350C_A		orf19.4906				0.99	0.97	-6.64	17	Uncharacterized ORF; Putative adhesin-like protein; positively regulated by Tbf1; Spider biofilm induced
C6_02680W_A		orf19.5539				0.68	0.85	-6.64	17	Uncharacterized ORF; Protein of unknown function
C6_02310W_A		orf19.3473				0.91	0.98	-6.64	17	Uncharacterized ORF; Protein of unknown function
C6_03370W_A		orf19.5626	Wh		Wh	0.83	0.66	-6.64	17	Uncharacterized ORF; Protein of unknown function; Plc1-regulated; induced by Mnl1 under weak acid stress; flow model biofilm induced
C1_01740W_A	CTN1	orf19.4551	Op	Op		2.52	0.97	-6.64	17	Carnitine acetyl transferase; required for growth on nonfermentable carbon sources, not for hyphal growth or virulence in mice; induced in macrophage; macrophage/pseudohyphal-repressed after 16 hr; rat catheter, Spider biofilm induced
CR_04820W_A		orf19.6311		Op	Wh	0.31	0.38	-6.64	17	Uncharacterized ORF; Protein of unknown function; Hap43-induced; rat catheter and Spider biofilm induced
CR_07450C_A	MNL1	orf19.6121				0.86	0.9	-6.64	17	Transcription factor; induces transcripts of stress response genes via SLE (STRE-like) elements; required for adaptation to weak acid stress; activates a subset of the genes that are repressed by Nrg1
C6_03330C_A		orf19.5621				0.79	0.86	-6.64	17	Putative protein of unknown function; mutation confers hypersensitivity to amphotericin B; overlaps orf19.5621
C5_03560W_A		orf19.6667				0.94	1.09	-6.64	17	Uncharacterized ORF; Predicted histone deacetylase activity; Spider biofilm induced
C1_08650C_A	FAD1	orf19.4723				1.08	1.26	-6.64	17	Uncharacterized ORF; Protein of unknown function
C4_06460C_A		orf19.2888				0.9	0.94	-6.64	17	Uncharacterized ORF; Protein of unknown function
C7_00830C_A		orf19.7034				0.98	1.09	-6.64	17	Uncharacterized ORF; Putative eIF4E-associated protein;, accelerates mRNA degradation by promoting decapping; Spider biofilm repressed
C4_04030W_A	JEN2	orf19.5307	Op	Op	Op	1.55	0.04	-6.64	17	Dicarboxylic acid transporter; regulated by glucose repression; induced by Rgt1; disruptants not obtained by UAU1 method; rat catheter and Spider biofilm induced
C5_02990W_A		orf19.4324				1.41	1.05	-6.64	17	Uncharacterized ORF; Protein of unknown function
C2_05780C_A	MKK2	orf19.6889				0.69	0.75	-6.64	17	Ortholog of S. cerevisiae Mkk2; MAP kinase kinase involved in signal transduction; macrophage-downregulated; mutants are viable and hypersensitive to caspofungin
C2_02620W_A		orf19.1588				0.94	0.83	-6.64	17	Uncharacterized ORF; Putative mitochondrial protein of unknown function; regulated by Sef1p-, Sfu1p-, and Hap43p
C4_02860W_A		orf19.2710				0.98	0.98	-6.64	17	Uncharacterized ORF; Protein of unknown function
C1_05500W_A	RSM22	orf19.414				1.67	1.64	-6.64	17	Uncharacterized ORF; Predicted mitochondrial small ribosomal subunit; rat catheter and Spider biofilm induced
C5_02110W_A		orf19.4216			Wh	0.08	3.12	-6.64	17	Uncharacterized ORF; Putative heat shock protein; decreased expression in hyphae; transcription is increased in populations of cells exposed to fluconazole over multiple generations; overexpression increases resistance to farnesol and azoles
			3 fase opac	4 fase opaca	2 fase opaca					
			1 fase blanc	0 fase blanca	5 fase blanca					
**Lower in WOR1^{OE}**										
C5_03290C_A		orf19.2650.1				1.12	1.03	-6.43	9.547141077	Uncharacterized ORF; Mitochondrial ribosomal protein of the small subunit; Spider biofilm repressed
C2_00250W_A	STF2	orf19.2107.1			Wh	0.74	0.79	-6.37	9.390839648	Uncharacterized ORF; Protein involved in ATP biosynthesis; repressed in hyphae; repressed by Efg1, Hap43; transcript upregulated in clinical isolates from HIV+ patients with oral candidiasis; rat catheter, flow model and Spider biofilm induced
C1_10740C_A	ASR1	orf19.2344	Wh		Wh	0.75	1.26	-6.23	7.769950679	Uncharacterized ORF; Heat shock protein; transcript regulated by cAMP, osmotic stress, ciclopirox olamine, ketoconazole; repressed by Cyr1, Ras1; colony morphology-related regulated by Ssn6; stationary phase enriched; Hap43-induced; Spider biofilm induced
C2_04010C_A	HSP21	orf19.822	Wh	Wh	Wh	0.84	1.13	-5.83	8.040698186	Small heat shock protein; role in stress response and virulence; fluconazole-downregulated; induced in cyr1 or ras1 mutant; stationary phase enriched protein; detected in some, not all, biofilm extracts; Spider biofilm induced
CR_05340C_A	IFE2	orf19.5288	Wh	Wh	Wh	0.06	0.72	-5.29	6.800121307	Uncharacterized ORF; Putative alcohol dehydrogenase; yeast-enriched transcript; Efg1-regulated; induced by prostaglandins, Hog1, fluconazole; rat catheter biofilm induced
C2_08290C_A	UCF1	orf19.1354			Wh	0.4	0.29	-5.01	6.207308499	Uncharacterized ORF; Upregulated by cAMP in filamentous growth; induced in high iron, decreased upon yeast-hypha switch; downregulation correlates with clinical fluconazole resistance; Ras1-regulated; Hap43-repressed; flow model biofilm induced
C7_00700W_A	CYB5	orf19.7049			Op	1.28	0.86	-4.91	5.991268315	Cytochrome b(5); ortholog of S. cerevisiae Cyb5; induced in high iron; fluconazole-induced; shows colony morphology-related gene regulation by Ssn6; mutants are viable
C4_01270W_A	RPS6A	orf19.4660				1.05	0.83	-4.72	5.612592817	Ribosomal protein 6A; localizes to cell surface of yeast cells but not hyphae; repressed upon phagocytosis by murine macrophage; possibly essential; Hap43-induced; Spider biofilm repressed
C5_02080C_A	HSP12	orf19.3160	Wh	Wh		0.08	2.58	-4.69	5.552610158	Uncharacterized ORF; Heat-shock protein; induced by osmotic/oxidative/cadmium stress, fluphenazine treatment, low iron, CDR1 and CDR2 overexpression, or ssn6 or ssk1 null mutation; overexpression increases resistance to farnesol and azoles
C1_00460W_A		orf19.6062				1.28	1.17	-4.64	5.441266543	Putative TIM23 translocase complex subunit; membrane-localized; Hap43-repressed
CR_08890C_A	ASR2	orf19.7284	Wh			0.40	1.07	-4.6	5.375273125	Uncharacterized ORF; Adenylyl cyclase and stress responsive protein; induced in cyr1 or ras1 mutant; stationary phase enriched protein; Spider biofilm induced
C1_12850W_A	BLP1	orf19.4914.1						-4.57	5.317166935	Uncharacterized ORF; Protein of unknown function, serum-induced
CR_00200W_A	PCK1	orf19.7514		Wh		0.73	0.35	-4.33	4.859528267	Uncharacterized ORF; Phosphoenolpyruvate carboxykinase; glucose, C-source, yeast-hypha, Hap43 regulated; fluconazole, phagocytosis, H2O2, oral candidasis, Spider/rat catheter/flow model biofilm induced; repressed in biofilm by Bcr1, Tec1, Ndt80, Rob1, Brg1
C6_01700W_A	RPL32	orf19.3415.1				0.99	0.92	-4.32	4.840366885	Component of the large (60S) ribosomal subunit; Spider biofilm repressed
C1_11200W_A		orf19.2296	Wh			0.91	1.06	-4.23	4.680279358	Uncharacterized ORF; Predicted mucin-like protein; ketoconazole-induced; fluconazole-repressed; induced in cyr1 mutant; colony morphology-related gene regulation by Ssn6; flow model biofilm induced; Spider biofilm induced
C7_02680W_A	TOM22	orf19.3696				1.58	1.49	-4.22	4.654495209	Putative mitochondrial import receptor subunit; colony morphology-related gene regulation by Ssn6
C3_04680W_A	RPP2B	orf19.5928				0.91	0.98	-4.16	4.542821063	Conserved acidic ribosomal protein; possibly involved in regulation of translation elongation; interacts with Rpp1A; 1 of 4 similar C. albicans proteins (Rpp1A, Rpp1B, Rpp2A, Rpp2B); macrophage/pseudohyphal-induced; Spider biofilm repressed
C2_08450W_A		orf19.3635				0.84	1.02	-4.12	4.486505945	Uncharacterized ORF; Protein of unknown function
C7_02170C_A		orf19.6498				1.48	1.41	-4.12	4.24437353	Uncharacterized ORF; Protein of unknown function
C2_08640C_A	PST2	orf19.3612		Op	Op	3.91	0.71	-4.09	3.860292353	Putative NADH:quinone oxidoreductase; similar to 1,4-benzoquinone reductase; immunogenic in mice; induced by benomyl, oxidative stress via Cap1; fungal-specific; farnesol-repressed; Spider biofilm induced
C5_02190C_A		orf19.4225.1						-3.87	4.040331963	Uncharacterized ORF; Protein of unknown function
C2_00760C_A		orf19.2048		Wh	Wh	0.64	3.48	-3.83	3.537096708	Uncharacterized ORF; Proten of unknown function; transcript positively regulated by Sfu1; Hap43 repressed; Spider biofilm induced
C1_04500W_A	ICL1	orf19.6844		Op	Op	1.84	1.04	-3.76	3.867465087	Isocitrate lyase; glyoxylate cycle enzyme; required for virulence in mice; induced upon phagocytosis by macrophage; farnesol regulated; Pex5-dependent peroxisomal localization; stationary phase enriched; rat catheter, Spider biofilm induced
C2_02260W_A		orf19.1544						-3.76	3.80566306	Uncharacterized ORF; Putative cis-golgi localized protein involved in ER to Golgi transport; Spider biofilm repressed
C5_01910W_A	GIS2	orf19.3182		Wh	Wh	0.64	1.04	-3.61	3.626116449	Translational activator for mRNAs with internal ribosome entry sites; induced in high iron; repressed by yeast-hypha switch; null exhibits sensitivity to sorbitol, 5-fluorocytosine, and cold temperatures; Spider biofilm repressed
C2_05610C_A	RPS8A	orf19.6873			Wh	0.83	1.75	-3.39	3.274929608	Small 40S ribosomal subunit protein; induced by ciclopirox olamine; repressed upon phagocytosis by murine macrophage; 5'-UTR intron; Hap43-induced; Spider biofilm repressed
C4_01150W_A	ASK1	orf19.4675				0.94	0.9	-3.39	2.954310886	Uncharacterized ORF; Essential subunit of the Dam1 (DASH) complex, which acts in chromosome segregation by coupling kinetochores to spindle microtubules
C7_03380W_A		orf19.1336.2				1.44	1.4	-3.37	3.170990101	Uncharacterized ORF; Protein of unknown function
C3_00480C_A	DOT5	orf19.5417				1.1	1.02	-3.34	3.212406609	Uncharacterized ORF; Putative nuclear thiol peroxidase; alkaline downregulated; sumoylation target; Spider and flow model biofilm induced
C2_03090C_A	ADE8	orf19.5789				0.95	1.02	-3.34	3.205835187	Putative phosphoribosylglycinamide formyl-transferase, enzyme of amino acid biosynthesis pathway; upregulated in biofilm; S. cerevisiae ortholog is Gcn4p regulated; protein enriched in stationary phase yeast-form cultures
C2_03790C_A	ASR3	orf19.842				1.28	1.46	-3.32	3.173349469	Uncharacterized ORF; Adenylyl cyclase and stress responsive protein; induced in cyr1 or ras1 mutant; Spider biofilm induced
C7_01610W_A		orf19.6563.1				1.26	1.26	-3.3	2.886374403	Uncharacterized ORF; Protein of unknown function
C3_06830C_A		orf19.6818				0.81	0.89	-3.3	3.058780402	Uncharacterized ORF; Protein of unknown function
C1_06470W_A		orf19.6264.4				1.29	1.32	-3.25	3.070303744	Uncharacterized ORF; Ortholog of S. cerevisiae Rpl39; a component of the 60S ribosomal subunit; Hap43-induced; Spider biofilm repressed
C1_03640C_A	HFL1	orf19.3063				1.49	1.68	-3.23	2.722610126	Uncharacterized ORF; HAP5-like; ortholog of S. cerevisiae Dpb3; third-largest subunit of DNA polymerase II (DNA polymerase epsilon); phosphorylated protein; mutants have a growth defect
C7_00860W_A	SSR1	orf19.7030				1.32	1.11	-3.22	2.931933459	Beta-glucan associated ser/thr rich cell-wall protein with a role in cell wall structure; GPI anchor; similar mRNA abundance in yeast-form and germ tubes; detected at germ tube plasma membrane; repressed in cells treated with Congo Red
C5_02700W_A	CTA7	orf19.4288				0.78	0.87	-3.21	2.743053859	Zn(II)2Cys6 transcription factor; activates transcription in 1-hybrid assay in S. cerevisiae; has similarity to S. cerevisiae Stb4
C4_00170W_A		orf19.5642				1.01	0.82	-3.16	2.941920465	Uncharacterized ORF; Ortholog of S. cerevisiae Dif1; which regulates nuclear localization of Rnr2 and Rnr4; colony morphology-related gene regulation by Ssn6; induced in cyr1 mutant; Spider biofilm induced
C2_07630C_A		orf19.1862	Wh	Wh	Wh	0.64	1.15	-3.13	2.903824863	Uncharacterized ORF; Possible stress protein; increased transcription associated with CDR1 and CDR2 overexpression or fluphenazine treatment; regulated by Sfu1, Nrg1, Tup1; stationary phase enriched protein; Spider biofilm induced

C6_02740W_A		orf19.5547				1.13	1.06	-3.1	2.852881194	Uncharacterized ORF; Protein of unknown function; Hap43-repressed gene
C4_03390W_A	CSH3	orf19.3366				1.41	0.92	-3.08	2.675686939	Functional homolog of S. cerevisiae Shr3p, which is a chaperone specific for amino acid permeases; localized to ER; required for wild-type amino-acid responsive hyphal growth and for mouse systemic virulence; regulated by Gcn2p and Gcn4p
C2_03590C_A	CAP4	orf19.861				1	0.98	-3.02	2.747471569	Uncharacterized ORF; Predicted bZip transcription factor; possibly an essential gene, disruptants not obtained by UAU1 method
CR_07230W_A		orf19.6147				1.05	0.86	-2.95	2.649492762	Uncharacterized ORF; Putative histone chaperone; role in chromatin remodeling; rat catheter and Spider biofilm repressed
C1_01980W_A	HGT1	orf19.4527				13.96	0.49	-2.95	2.647725931	High-affinity MFS glucose transporter; induced by progesterone, chloramphenicol, benomyl; likely essential for growth; protein newly produced during adaptation to the serum; rat catheter and Spider biofilm induced
C1_02130C_A	GAL1	orf19.3670				2.49	1.19	-2.86	2.52718008	Galactokinase; galactose, Mig1, Tup1, Hap43 regulated; fluconazole, ketoconazole-induced; stationary phase enriched protein; GlcNAc-induced protein; farnesol, hypoxia-repressed in biofilm; rat catheter and Spider biofilm induced
CR_09140C_A		orf19.7310				0.29	0.84	-2.83	2.489434101	Uncharacterized ORF; Protein with a role in directing meiotic recombination events to homologous chromatids; induced by ciclopirox olamine; positively regulated by Sfu1; Hog1, fluconazole-repressed; Hap43-induced; Spider biofilm induced
C1_02510W_A		orf19.2940				1.37	1.27	-2.82	2.319349442	Uncharacterized ORF; Putative v-SNARE of the endoplasmic reticulum membrane; possibly an essential gene, disruptants not obtained by UAU1 method
C6_01070C_A	CIP1	orf19.113				0.91	0.98	-2.8	2.455730095	Possible oxidoreductase; transcript induced by cadmium but not other heavy metals, heat shock, yeast-hypha switch, oxidative stress (via Cap1), or macrophage interaction; stationary phase enriched protein; Spider biofilm induced
C5_03360W_A	RPO26	orf19.2643				0.78	1.02	-2.79	2.435895746	Putative RNA polymerase subunit; heterozygous null mutant exhibits resistance to parnafungin in the C. albicans fitness test
C6_03770C_A	ORM1	orf19.5751				0.91	0.95	-2.78	2.32486792	Putative endoplasmic reticulum membrane protein; Hap43p-repressed gene; mutation confers hypersensitivity to aureobasidin A
C1_00650C_A	SNF7	orf19.6040				1.38	1.29	-2.77	2.408788773	ESCRT III complex protein; role in proteolytic activation of Rim101 and Rim8 processing/activation; separable roles in RIM101 pathway and in transport from MVB to vacuole; involved in echinocandin and azole sensitivity
C3_00220W_A	HGT19	orf19.5447				1.4	0.62	-2.74	2.296367014	Putative MFS glucose/myo-inositol transporter; 20 member family; 12 transmembrane segments, extended N terminus; expressed in rich medium; Hap43, phagocytosis, rat catheter, Spider and flow model biofilm induced
C4_02290W_A	LAB5	orf19.2774				0.94	0.93	-2.67	2.104306503	Uncharacterized ORF; Protein of unknown function
C7_00160C_A		orf19.7107			Wh	1.41	1.71	-2.61	2.172652513	Uncharacterized ORF; Protein of unknown function
CR_02690W_A		orf19.2828				0.89	0.84	-2.56	2.150077368	Uncharacterized ORF; Protein of unknown function
C1_14110C_A	RPL4B	orf19.7217				0.96	1.08	-2.56	2.149893897	Ribosomal protein 4B; repressed upon phagocytosis by murine macrophage; Spider biofilm repressed
C2_00210W_A	RPL38	orf19.2111.2				0.96	0.89	-2.53	2.117089787	Uncharacterized ORF; 60S ribosomal ribosomal protein subunit; genes encoding cytoplasmic ribosomal subunits, translation factors, tRNA synthetases are downregulated upon phagocytosis by murine macrophage
C3_01550C_A	TOS1	orf19.1690				0.9	1.05	-2.49	1.992396914	Protein similar to alpha agglutinin anchor subunit; secreted; exogenously expressed protein is a substrate for Kex2 processing in vitro; fluconazole-induced; induced by alpha pheromone in SpiderM medium; Hap43-induced
C7_03630C_A	TIM9	orf19.6696				1.6	1.62	-2.48	2.060083116	Uncharacterized ORF; Predicted protein of the mitochondrial intermembrane space; rat catheter biofilm induced; Spider biofilm repressed
C3_07310C_A	SLK19	orf19.6763				0.84	0.73	-2.46	2.03308778	Alkaline-induced protein of plasma membrane; affects cell aggregation, cell wall; similar to S. cerevisiae Slk19p (a kinetochore protein with roles in mitosis, meiosis); required for wild-type virulence in mouse; macrophage-downregulated
C5_04880C_A	PUT2	orf19.3974				1	0.91	-2.45	2.01316464	Uncharacterized ORF; Putative delta-1-pyrroline-5-carboxylate dehydrogenase; alkaline upregulated; protein present in exponential and stationary growth phase yeast cultures; flow model biofilm induced; Spider biofilm induced
C4_02480C_A	TIM13	orf19.2754				1.12	0.93	-2.44	2.005382529	Uncharacterized ORF; Predicted mitochondrial intermembrane space protein with a role in protein import into mitochondria
C2_01740C_A		orf19.1485				1.06	0.95	-2.41	1.977212614	Uncharacterized ORF; Mitochondrial ribosomal protein of the large subunit; rat catheter biofilm induced
CR_05170C_A	FDH1	orf19.638	Op		Op	13.38	0.22	-2.41	1.966578543	Uncharacterized ORF; Formate dehydrogenase; oxidizes formate to CO2; Mig1 regulated; induced by macrophages; fluconazole-repressed; repressed by Efg1 in yeast, not hyphal conditions; stationary phase enriched; rat catheter and Spider biofilm induced
C6_03820C_A		orf19.5757				0.75	0.82	-2.4	1.889364503	Uncharacterized ORF; Protein of unknown function
C1_09860C_A	TFA1	orf19.4851				1.54	1.63	-2.4	1.859158194	Uncharacterized ORF; Protein with polyglutamate motifs and abundant Ser/Thr residues; described as a subunit of TFIIE, which is a basal transcription initiation factor of RNA Polymerase II; possibly an essential gene, disruptants not obtained by UAU1 method
C4_00770C_A		orf19.4164				0.95	0.91	-2.39	1.953489515	Uncharacterized ORF; Protein of unknown function
C3_05710W_A	RCT1	orf19.7350				0.79	1.36	-2.39	1.951689522	Uncharacterized ORF; Fluconazole-induced protein; Ras1, Cyr1 repressed and Efg1 induced; regulated by Nrg1, Tup1, Tbf1, Ssn6; induced in oralpharyngeal candidasis; rat catheter biofilm repressed
C6_00930C_A		orf19.94		Op	Op	1.37	0.74	-2.38	1.941176122	Uncharacterized ORF; Protein of unknown function; Spider biofilm induced
C3_02920W_A		orf19.285				1.18	0.9	-2.38	1.932464838	Uncharacterized ORF; Ortholog of S. cerevisiae Igo2; role in mRNA stabilization of mRNA; Spider biofilm induced
C1_07680W_A	HAP2	orf19.1228				1.21	1.14	-2.37	1.846368636	CCAAT-binding transcription factor; regulates low-iron induction of FRP1; in these conditions CBF comprises Hap43 and probably Hap2 and Hap3; possibly essential, disruptants not obtained by UAU1 method; Cap1-dependent induction in low iron
C2_04600C_A	RPL17B	orf19.4490				0.89	0.84	-2.36	1.918758858	Ribosomal protein L17; mutation confers hypersensitivity to 5-FU, tubercidin; repressed upon phagocytosis by macrophage; Hap43-induced; Spider biofilm repressed
C4_06030W_A	CMD1	orf19.4413				0.96	1.07	-2.34	1.896775553	Calmodulin; calmodulin inhibitors cause a defect in hyphal growth; transcript not regulated by yeast-hyphal transition; mutation confers hypersensitivity to cytochalasin D and high concentrations of tunicamycin; gene has intron
C5_00310C_A		orf19.962				1.11	1.19	-2.34	1.89577012	Uncharacterized ORF; Protein with a fungal RNA polymerase I subunit RPA14 domain; proposed to play a role in the recruitment of pol I to the promoter; Hap43-induced gene
C2_04540C_A	GDT1	orf19.4496				0.75	0.98	-2.33	1.77343337	Uncharacterized ORF; Protein of unknown function
C3_02610C_A	GLX3	orf19.251			Op	3.07	1.46	-2.33	1.877156253	Glutathione-independent glyoxalase; binds human immunoglobulin E; alkaline, fluconazole, Hog1 repressed; hypoxia, oxidative stress via Cap1, Hap43 induced; stationary-phase enriched; rat catheter, Spider biofilm induced
C3_04920C_A		orf19.5961				1.11	0.9	-2.32	1.868767174	Uncharacterized ORF; Protein of unknown function
CR_01390W_A	MGE1	orf19.2524				0.88	1.03	-2.31	1.856368651	Uncharacterized ORF; Putative mitochondrial matrix cochaperone; macrophage/pseudohyphal-repressed
CR_04580W_A		orf19.549				1.16	1.06	-2.3	1.830595384	Uncharacterized ORF; Protein of unknown function
C1_01040W_A		orf19.3292				1.37	1.62	-2.3	1.788618641	Uncharacterized ORF; Protein of unknown function
C7_03860W_A		orf19.7196				1.13	1.5	-2.3	1.842597952	Uncharacterized ORF; Putative vacuolar protease; upregulated in the presence of human neutrophils; Spider biofilm induced
C3_03340C_A		orf19.332.1						-2.3	1.842083302	Uncharacterized ORF; Protein of unknown function
C1_11670W_A		orf19.1152	Wh		Wh	0.5	2.72	-2.29	1.785822699	Uncharacterized ORF; Protein of unknown function; induced in core stress response; Gcn2 and Gcn4 regulated; flow model biofilm induced; Spider biofilm induced
C1_09710C_A		orf19.4835				1.57	1.67	-2.29	1.774046372	Uncharacterized ORF; Protein of unknown function
CR_03030C_A	RPL28	orf19.2864.1				0.68	0.77	-2.27	1.807529654	Uncharacterized ORF; Putative ribosomal protein; Plc1-regulated; downregulated upon phagocytosis by murine macrophage; Spider biofilm repressed
C1_12250C_A		orf19.5238				1.5	1.11	-2.26	1.780349808	Uncharacterized ORF; Protein of unknown function
C6_03880W_A		orf19.5763				1.33	0.97	-2.26	1.795857597	Uncharacterized ORF; Protein of unknown function
CR_09650W_A	WAL1	orf19.6598				0.77	0.88	-2.23	1.66085678	Protein required for hyphal growth and for wild-type cell morphology, polarized budding, endocytosis, vacuole morphology; similar to Wiskott-Aldrich syndrome protein; localizes to cortical actin patches and hyphal tips
C2_01550W_A	SMP2	orf19.1462				0.85	0.98	-2.22	1.75746209	Uncharacterized ORF; Putative Mg2+-dependent phosphatidate phosphatase; transcript regulated by Nrg1
C1_07510W_A		orf19.2794				1.39	1.39	-2.22	1.757529711	Uncharacterized ORF; Putative non-specific single-domain racemase; regulated by Gcn4p; repressed in response to amino acid starvation (3-AT treatment); alkaline upregulated; macrophage-induced protein
C1_03150C_A		orf19.3007.2			Wh	0.66	1.78	-2.22	1.682680538	Uncharacterized ORF; Protein of unknown function
CR_09190C_A		orf19.7316			Op	1.07	0.98	-2.22	1.653529647	Putative phytanoyl-CoA dioxygenase family protein; mutation confers hypersensitivity to 5-fluorocytosine (5-FC), 5-fluorouracil (5-FU), and tubercidin (7-deazaadenosine); induced by nitric oxide
C1_06060C_A	ACP1	orf19.2439				1.29	1.28	-2.21	1.751202856	Uncharacterized ORF; Fatty acid biosynthetic protein; shows colony morphology-related gene regulation by Ssn6p; protein newly produced during adaptation to the serum
C2_05710C_A		orf19.6882.1				0.9	0.84	-2.21	1.748637569	Uncharacterized ORF; Ribosomal 60S subunit protein; Spider biofilm repressed
C2_04820W_A		orf19.182				0.88	1.12	-2.21	1.671655315	Uncharacterized ORF; Protein of unknown function
C4_04890C_A	RPL24A	orf19.3789				1.02	0.94	-2.21	1.741646266	Uncharacterized ORF; Predicted ribosomal protein; downregulated upon phagocytosis by murine macrophage; intron in 5'-UTR; Hap43-induced; Spider biofilm repressed
C4_02940W_A	ABP1	orf19.2699				1.08	0.72	-2.17	1.707748246	Ortholog of S. cerevisiae Abp1; actin-binding protein of the cortical actin cytoskeleton; caspofungin induced; protein only detected in stationary phase yeast-form cultures; Spider biofilm repressed
C4_06470W_A		orf19.2887				1.46	1.25	-2.16	1.69600006	Uncharacterized ORF; Ortholog in S. cerevisiae is localizedto the bud, mating projection tip, and associates with ribosomes; Spider biofilm induced
CR_02070C_A	ADH5	orf19.2608	Wh	Wh		1.17	0.64	-2.16	1.690724831	Putative alcohol dehydrogenase; regulated by white-opaque switch; fluconazole-induced; antigenic in murine infection; regulated by Nrg1, Tup1; Hap43, macrophage repressed, flow model biofilm induced; Spider biofilm induced
C3_04670C_A	RPS15	orf19.5927				1.05	1	-2.16	1.690454341	Uncharacterized ORF; Putative ribosomal protein; macrophage/pseudohyphal-induced after 16 h; repressed upon phagocytosis by murine macrophage; Spider biofilm repressed
CR_00350W_A		orf19.7499				0.66	0.82	-2.16	1.68980897	Uncharacterized ORF; Putative nicotinic acid mononucleotide adenylyltransferase, involved in NAD salvage pathway; Spider biofilm repressed
C2_07200W_A		orf19.2276				0.84	0.66	-2.15	1.657410478	Uncharacterized ORF; Protein of unknown function
C5_03830C_A		orf19.1111				0.97	1.07	-2.15	1.655253109	Uncharacterized ORF; Protein of unknown function
C3_05780C_A	CRZ1	orf19.7359	Wh		Wh	0.88	1.35	-2.15	1.63513503	Calcineurin-regulated C2H2 transcription factor; role in maintenance of membrane integrity, azole tolerance; not required for mouse virulence; repressed by low iron; regulates Ca++ influx during alkaline pH response; Spider biofilm induced
C3_04490W_A	RAX1	orf19.5903			Op	0.9	0.67	-2.15	1.580754215	Uncharacterized ORF; Protein with a predicted role in bud site selection; hypha-induced expression; Spider biofilm induced
C3_05420W_A		orf19.6980				0.75	0.9	-2.14	1.623652522	Uncharacterized ORF; Protein of unknown function
C4_05610C_A		orf19.1246	Wh			0.8	1.11	-2.12	1.644507835	Uncharacterized ORF; Putative eisosome component role in proper eisosome assembly; upregulated in cyr1 null mutant
C1_09350W_A		orf19.4796				1.41	1.37	-2.11	1.633514049	Uncharacterized ORF; Putative eIF-4E-binding repressor of CAP-dependent translation; stationary phase enriched protein
C1_10090C_A		orf19.4878				1.24	1.23	-2.11	1.612924201	Uncharacterized ORF; Protein of unknown function; Hap43-repressed; rat catheter biofilm repressed
C3_01890C_A		orf19.1658				1.04	0.9	-2.1	1.626877638	Uncharacterized ORF; Protein of unknown function
C7_00800C_A	YAE1	orf19.7037				1.18	1.14	-2.09	1.601254884	Uncharacterized ORF; Protein of unknown function
C3_04500C_A	RPL19A	orf19.5904				0.86	0.88	-2.08	1.611621379	Ribosomal protein L19; repressed upon phagocytosis by murine macrophages; Hap43-induced gene; Spider biofilm repressed
CR_04360C_A	NHP2	orf19.526				0.81	1.02	-2.08	1.581609114	Uncharacterized ORF; Putative H/ACA snoRNP protein; macrophage/pseudohyphal-induced
C1_02060W_A		orf19.3663.1				1.19	1.13	-2.08	1.573935186	Uncharacterized ORF; Predicted plasma membrane protein; gene has intron
C7_00330C_A		orf19.7088				1.46	1.61	-2.07	1.511141987	Uncharacterized ORF; Protein of unknown function
C7_03180C_A	HHO1	orf19.5137.1			Wh	1.01	1.28	-2.07	1.593343615	Uncharacterized ORF; Putative histone H1; farnesol regulated; Hap43-induced; contains 5' UTR intron; Spider biofilm repressed
C2_08180C_A		orf19.2167				1.1	1.14	-2.06	1.543365786	Uncharacterized ORF; Protein of unknown function
C3_04910C_A	NCE102	orf19.5960			Wh	0.79	0.82	-2.05	1.576287432	Non classical protein export protein; localized to plasma membrane; Hap43-induced gene; flow model biofilm induced; Spider biofilm induced
C4_00600C_A	MUC1	orf19.4183				1.07	1.09	-2.05	1.561081114	Uncharacterized ORF; Protein similar to S. cerevisiae Muc1; a cell surface glycoprotein involved in filamentous growth; repressed by benomyl treatment; mutant is viable
C4_03960W_A		orf19.787.1						-2.04	1.491472293	Uncharacterized ORF; Protein of unknown function; ORF added to Assembly 21 based on comparative genome analysis; protein detected by mass spec in stationary phase cultures
C5_03540C_A	RPS25B	orf19.6663				0.97	1.01	-2.04	1.561625222	Uncharacterized ORF; Ribosomal protein; macrophage/pseudohyphal-induced after 16 h; repressed upon phagocytosis by murine macrophage; transcript positively regulated by Tbf1; 5'-UTR intron; Hap43-induced; Spider biofilm repressed
C5_04870W_A		orf19.3973				1.06	0.8	-2.03	1.527615737	Uncharacterized ORF; Ortholog of Hua1, a zinc finger domain protein with sequence similarity to Type I J-proteins; flow model biofilm induced
C1_06810W_A	CAT1	orf19.6229			Op	3.56	1.18	-2.03	1.553723788	Catalase; resistance to oxidative stress, neutrophils, peroxide; role in virulence; regulated by iron, ciclopirox, fluconazole, carbon source, pH, Rim101, Ssn6, Hog1, Hap43, Sfu1, Sef1, farnesol, core stress response; Spider biofilm induced
C2_02770W_A		orf19.5828				0.81	1	-2.03	1.529404271	Uncharacterized ORF; Protein of unknown function
C2_04960C_A		orf19.3528				0.85	1.02	-2.02	1.546723896	Uncharacterized ORF; Protein of unknown function; Spider biofilm induced
C2_01720C_A		orf19.1483				1.25	1.21	-2.02	1.539483948	Uncharacterized ORF; Protein of unknown function
C2_08310W_A	TIM22	orf19.1352				1	0.77	-2.01	1.502579909	Mitochondrial inner membrane protein; predicted role in protein import; Hap43-repressed gene; flow model biofilm induced; Spider biofilm repressed
C1_05160C_A		orf19.449			Wh	2.56	1.48	-2	1.526025964	Uncharacterized ORF; Putative phosphatidyl synthase; stationary phase enriched protein; transcript repressed by yeast-hypha switch; Hap43-repressed; rat catheter, Spider and flow model biofilm induced
C4_01700C_A	NHP6A	orf19.4623.3				0.96	1.24	-2	1.525147713	Putative non-histone chromatin component; RNA abundance regulated by tyrosol,cell density; Hap43-induced; amphotericin B, caspofungin repressed; 5'-UTR intron; rat catheter and Spider biofilm repressed

CR_02650C_A	DRE2	orf19.2825				0.71	0.83	-2	1.501308421	Uncharacterized ORF; Putative cytosolic Fe-S protein assembly protein; a-specific transcript; regulated by Sef1, Sfu1, and Hap43; rat catheter and Spider biofilm induced
C1_06840C_A		orf19.6225.1				1.56	1.15	-1.99	1.485298867	Uncharacterized ORF; Protein of unknown function
C2_05180W_A	WH11	orf19.3548.1			Wh	0.06	3.56	-1.99	1.448180195	White-phase yeast transcript; expression in opaques increases virulence/switching; mutant switches as WT; Hap43, hypoxia, ketoconazol induced; required for RPMI biofilm; Bcr1-induced in RPMI a/a biofilm; rat catheter, Spider biofilm induced
C1_06450C_A	RPS14B	orf19.6265.1				1.72	1.64	-1.98	1.500192482	Uncharacterized ORF; Putative ribosomal protein; repressed upon phagocytosis by murine macrophage; transcript positively regulated by Tbf1; Spider biofilm repressed
C3_07340W_A	GCY1	orf19.6757				1.67	0.96	-1.97	1.496673453	Aldo/keto reductase; mutation confers hypersensitivity to toxic ergosterol analog; farnesol-repressed; stationary phase enriched protein; flow model biofilm induced; Spider biofilm repressed
C2_01620W_A	COX4	orf19.1471				0.72	0.71	-1.97	1.495980911	Putative cytochrome c oxidase subunit IV; Mig1-regulated; macrophage/pseudohyphal-induced gene; macrophage-induced protein; repressed by nitric oxide; 5'-UTR intron; Hap43-repressed
C1_02310C_A		orf19.3688				1.1	1.15	-1.97	1.488064041	Uncharacterized ORF; Protein of unknown function
C4_06580W_A	CBF1	orf19.2876			Wh	0.67	0.89	-1.96	1.480680699	Transcription factor; binds ribosomal protein gene promoters and rDNA locus with Tbf1; regulates sulfur starvation-response, respiratory, glycolytic genes; does not bind to centromeres as does S. cerevisiae Cbf1; Spider biofilm repressed
C7_00720W_A	RTF1	orf19.7047				1.46	1.18	-1.94	1.436003563	Uncharacterized ORF; Putative RNA polymerase II-associated Paf1 complex subunit; induced during the mating process
C2_09830C_A		orf19.1367.1						-1.93	1.427159162	Uncharacterized ORF; Protein of unknown function
C2_02740C_A	HPT1	orf19.5832			Wh	0.47	0.81	-1.92	1.439321347	Uncharacterized ORF; Putative hypoxanthine-guanine phosphoribosyltransferase; protein abundance affected by URA3 expression in the CAI4 strain background; protein level decreases in stationary phase; Spider biofilm induced
C2_01610C_A	RPS26A	orf19.1470				1.05	1.02	-1.91	1.436678101	Uncharacterized ORF; Ribosomal protein; regulated by Nrg1, Tup1; repressed upon phagocytosis by murine macrophage; alternatively spliced intron in 5'-UTR; Spider biofilm repressed
C1_10970W_A		orf19.2319	Wh			1.4	1.36	-1.91	1.36940402	Uncharacterized ORF; Putative nucleolar protein with a predicted role in pre-rRNA processing; Hap43-induced gene; repressed in core stress response
CR_08460W_A	TPM2	orf19.6414.3				1.21	0.85	-1.89	1.412267356	Uncharacterized ORF; Putative tropomyosin isoform 2; regulated by Gcn4; repressed by amino acid starvation; macrophage-induced; protein levels decrease in stationary cells; Hap43-induced; rat catheter and Spider biofilm repressed
C1_12590W_A	ATP20	orf19.3757				1.34	1.33	-1.88	1.403007962	Putative mitochondrial ATP synthase; shows colony morphology-related gene regulation by Ssn6p; flucytosine induced; caspofungin repressed; macrophage/pseudohyphal-induced
C3_00090W_A	RPS24	orf19.5466				0.93	1.13	-1.88	1.402469952	Uncharacterized ORF; Predicted ribosomal protein; hyphal downregulated; repressed upon phagocytosis by murine macrophage; transcriptionally activated by Tbf1; Spider biofilm repressed
C1_06640C_A		orf19.6247.1						-1.88	1.349402786	Uncharacterized ORF; Protein of unknown function
C1_07080W_A		orf19.6196			Op	4.53	1.12	-1.87	1.370234344	Uncharacterized ORF; Component of the SWI/SNF and RSC chromatin remodeling complexes; suggested role in chromosome maintenance; Spider biofilm induced
C2_06890C_A		orf19.2244				0.69	1.85	-1.86	1.382228557	Uncharacterized ORF; Similar to oxidoreductases and to S. cerevisiae Yjr096wp; Sfu1 repressed; induced by benomyl treatment, Ssr1; Hap43-repressed; flow model biofilm repressed
C2_04190C_A	UGA1	orf19.802			Op	1.17	0.62	-1.86	1.379758225	Uncharacterized ORF; Putative GABA transaminase; transcription regulated by Mig1 and Tup1; stationary phase enriched protein; rat catheter and Spider biofilm induced
C1_00270W_A		orf19.6079			Op	0.88	0.46	-1.85	1.370519989	Uncharacterized ORF; Predicted ORF in retrotransposon Tca8 with similarity to the Gag region encoding nucleocapsid-like protein; repressed by ciclopirox olamine; filament induced; regulated by Rfg1, Tup1; overlaps orf19.6078.1
C3_05440C_A		orf19.6982				0.8	0.93	-1.84	1.33752938	Uncharacterized ORF; Protein of unknown function
C2_09260C_A	GLO2	orf19.4088				0.82	1	-1.83	1.357100935	Uncharacterized ORF; Protein of unknown function
CR_01060W_A	LAG1	orf19.3249				0.68	0.73	-1.83	1.334773736	Putative ceramide synthase component; Hap43p-repressed gene; amphotericin B repressed
C4_06350C_A	NUP60	orf19.2901				0.77	0.92	-1.82	1.34254479	Uncharacterized ORF; Ortholog of S. cerevisiae Nup60p; a subunit of the nuclear pore complex; mutants are viable
C2_03260W_A		orf19.904				0.61	0.72	-1.81	1.312559174	Uncharacterized ORF; Protein of unknown function
C2_08120W_A	MAF1	orf19.2173	Wh		Wh	0.69	0.8	-1.81	1.308772005	Uncharacterized ORF; Putative negative regulator of RNA polymerase III; decreased expression in hyphae vs yeast cells; caspofungin repressed; Spider biofilm repressed
C7_01070C_A		orf19.7006				1.76	1.24	-1.8	1.326063523	Uncharacterized ORF; Protein of unknown function
CR_09640C_A		orf19.6599.1				0.83	0.89	-1.79	1.302151874	Uncharacterized ORF; Protein of unknown function
C2_03380W_A	PAN1	orf19.886				0.76	0.78	-1.79	1.314246315	Essential protein involved in endocytosis and polarized growth; ortholog of S. cerevisiae Pan1, which is a part of a complex that regulates actin cytoskeleton; Spider biofilm repressed
CR_01470W_A	CSP37	orf19.2531			Wh	0.45	1.64	-1.79	1.313153856	Hyphal cell wall protein; role in progression of mouse systemic infection; predicted P-loop, divalent cation binding, N-glycosylation sites; expressed in yeast and hyphae; hyphal downregulated; stationary-phase enriched; GlcNAc-induced
C1_12390C_A	RPL27A	orf19.5225.2				1.9	1.86	-1.78	1.311237103	Ribosomal protein L27; Spider biofilm repressed
C4_01840C_A		orf19.4609				0.92	0.9	-1.78	1.304582402	Uncharacterized ORF; Putative dienelactone hydrolase; protein abundance is affected by URA3 expression in the CAI-4 strain background; protein present in exponential and stationary growth phase yeast cultures; rat catheter biofilm repressed
C1_03090W_A	RPS1	orf19.3002				1.26	1.18	-1.78	1.303466835	Uncharacterized ORF; Putative ribosomal protein 10 of the 40S subunit; elicits host antibody response during infection; transcript induced during active growth; Spider biofilm repressed
			1 fase opaca	3 fase opaca	12 fase opaca					
			13 fase blanca	8 fase blanca	21 fase blanca					
**Higher in WOR1^{OE}**										
C4_03800C_A	NUP84	orf19.1298				0.85	0.69	2.49	1.304031674	Uncharacterized ORF; Protein of unknown function
C4_06600W_A	TOP2	orf19.2873				1.05	1.01	2.81	1.303092455	DNA topoisomerase II; catalyzes ATP-dependent DNA relaxation and decatenation in vitro; Y842 predicted to be catalytic; functional homolog of S. cerevisiae Top2p; sensitive to amsacrine or doxorubicin; farnesol-upregulated in biofilm
CR_04910W_A	CDC43	orf19.1803				1	0.88	3.13	1.97546115	Beta subunit of heterodimeric protein geranylgeranyltransferase type I; GGTase I enzyme binds zinc, is Mg-dependent; Cdc42p is GGTase I substrate
CR_07810W_A	YHB5	orf19.3710				0.88	0.93	3.18	1.878741522	Uncharacterized ORF; Flavohemoglobin-related protein; not required for normal NO resistance; predicted globin/FAD-binding/NAD(P)-binding domains but lacks some conserved residues of flavohemoglobins; filament induced; rat catheter and Spider biofilm induced
C4_05060W_A	CDL1	orf19.3773				0.82	1.02	3.18	1.564428745	Uncharacterized ORF; Putative RNAse III, ortholog of S. cerevisiae RNT1; merged with orf19.3772 in Assembly 21
CR_00920W_A	CCE1	orf19.3264				0.94	0.76	3.39	1.60327555	Uncharacterized ORF; Putative Holliday junction resolving enzyme; similar to S. cerevisiae Cce1p
C2_04330C_A		orf19.4521				0.79	0.79	3.5	1.981598661	Uncharacterized ORF; S. cerevisiae ortholog Env9 has similarity to oxidoreductases and is proposed to have vacuolar functions, found in lipid particles; hyphal-induced expression
CR_01380W_A		orf19.2521				0.82	1.09	3.8	2.15878153	Uncharacterized ORF; Protein of unknown function
C4_06670W_A		orf19.3142				0.61	0.66	4.07	2.801440438	Uncharacterized ORF; Protein of unknown function
CR_07220C_A		orf19.6148				1.09	0.71	4.82	4.564387797	Uncharacterized ORF; Homolog of nuclear distribution factor NudE, NUDEL; regulates dynein targeting to microtubule plus ends; flow model biofilm repressed
**Only in WOR1^{OE}**										
C2_08860W_A		orf19.223				0.73	1.13	6.64	17	Uncharacterized ORF; Putative serine/threonine protein kinase; Hap43-repressed; induced by prostaglandins; possibly an essential gene, disruptants not obtained by UAU1 method; flow model biofilm induced; Spider biofilm induced
C5_05470W_A	EST1	orf19.4045				0.86	0.9	6.64	17	Telomerase subunit; allosteric activator of catalytic activity, but not required for catalytic activity; has TPR domain
CR_10190C_A		orf19.7595				0.92	0.86	6.64	17	Uncharacterized ORF; Protein of unknown function
CR_01230C_A	CDC27	orf19.3231				1.04	0.95	6.64	17	Uncharacterized ORF; Putative ubiquitin-protein ligase; periodic mRNA expression, peak at cell-cycle S/G2 phase
C2_10720C_A		orf19.5352				1.16	0.98	6.64	17	Uncharacterized ORF; Protein with a predicted magnesium transporter domain; mutants are viable
CR_02560C_A	ASG1	orf19.166				0.71	1	6.64	17	Gal4p family zinc-finger transcription factor with similarity to S. cerevisiae Asg1p
CR_00210W_A	ALK2	orf19.7513	Op	Op		1.58	0.37	6.64	17	Uncharacterized ORF; N-Alkane inducible cytochrome P450
C5_03300C_A		orf19.2650				0.91	1.11	6.64	17	Uncharacterized ORF; Protein of unknown function; rat catheter biofilm induced
C1_04980C_A		orf19.52				1.1	0.93	6.64	17	Uncharacterized ORF; Protein of unknown function
C1_14250C_A		orf19.7235			Wh	1.13	1.27	6.64	17	Putative protein of unknown function; mutation confers hypersensitivity to amphotericin B
C1_10660W_A	TBP1	orf19.1837				1.38	1.62	6.64	17	ranscription initiation factor; binds TATA box sequence, binding does not require TFIIA; caspofungin repressed; functional homolog of S. cerevisiae and human TATA-binding proteins; Spider biofilm induced
C3_02260C_A		orf19.1619				0.84	0.99	6.64	17	Uncharacterized ORF; Putative kinase subunit of RNA polymerase II carboxy-terminal domain kinase I; possibly an essential gene, disruptants not obtained by UAU1 method
C2_07660W_A	PDS5	orf19.2216				0.82	0.99	6.64	17	Uncharacterized ORF; Putative protein with a predicted role in establishment and maintenance of sister chromatid condensation and cohesion; cell-cycle regulated periodic mRNA expression
C3_05810C_A	SKN1	orf19.7362				0.79	1.06	6.64	17	Uncharacterized ORF; Protein with a role in beta-1,6-glucan synthesis; probable N-glycosylated type II membrane protein; transcript and mRNA length change induced by yeast-hypha transition; induced by Rim101, caspofungin; rat catheter and Spider biofilm induced
C5_04520W_A	TIP41	orf19.3937				0.85	0.88	6.64	17	Uncharacterized ORF; Protein of unknown function
CR_02370W_A	ERG25	orf19.3732				1.04	0.74	6.64	17	Uncharacterized ORF; Putative C-4 methyl sterol oxidase; C4-demethylation of ergosterol biosynthesis intermediates, based on similarity to S. cerevisiae Erg25; fluconazole-induced; induced in azole-resistant strain; rat catheter and Spider biofilm induced
C6_02420W_A		orf19.3483	Op		Op	1.29	0.83	6.64	17	Putative phosphatidyl glycerol phospholipase C; Plc1-regulated; flow model biofilm induced; Spider biofilm induced
C1_07660W_A		orf19.2778				1.11	1.46	6.64	17	Uncharacterized ORF; Protein of unknown function; transcript is upregulated in clinical isolates from HIV+ patients with oral candidiasis
C3_00780W_A	HOS2	orf19.5377				0.84	0.97	6.64	17	Putative histone deacetylase; mutations affect filamentous growth; genetic evidence suggests that Set3p and Hos2p function together as a complex involved in regulation of white-opaque switching, morphogenesis, and virulence
C5_02670W_A	BUR2	orf19.4284				0.78	0.88	6.64	17	Uncharacterized ORF; Protein with similarity to S. cerevisiae Bur2p, contains a cyclin domain; not required for wild-type hyphal growth, adherence to buccal epithelial cells, or virulence in mouse systemic infection
C1_04530C_A		orf19.6847				1.39	1.4	6.64	17	Uncharacterized ORF; Putative tRNA U44 2'-O-methyltransferase; virulence-group-correlated expression; induced during oral infection; mutants have reduced ability to damage oral epithelial cells; early-stage flow model biofilm induced
C5_01280C_A	NUF2	orf19.1941				1.62	1.37	6.64	17	Kinetochore component; amount of Nuf2p and Mtw1p protein detected at each centromere is consistent with a single kinetochore microtubule attachment site
C4_06340W_A	AGO1	orf19.2903			Op	1.09	0.66	6.64	17	Putative Argonaute protein involved in RNA silencing; hyphal-induced expression; regulated by Cyr1, Ras1, Efg1, Nrg1, Tup1; rat catheter biofilm repressed
C6_04610C_A	NAG3	orf19.2158			Wh	0.27	0.46	6.64	17	Putative MFS transporter; similar to Nag4; required for wild-type mouse virulence and cycloheximide resistance; in gene cluster that includes genes encoding enzymes of GlcNAc catabolism; Spider biofilm repressed
CR_04870C_A		orf19.6306			Op	1.47	0.68	6.64	17	Trimethylaminobutyraldehyde dehydrogenase, the third enzyme of the carnitine biosynthesis pathway
CR_09410W_A		orf19.7337				0.82	0.91	6.64	17	Uncharacterized ORF; Protein with a nischarin related domain and leucine rich repeats; Spider biofilm induced
C1_14330W_A		orf19.7245				1.21	1.35	6.64	17	Uncharacterized ORF; Protein of unknown function
C2_09710C_A		orf19.1381				0.72	0.78	6.64	17	Uncharacterized ORF; Ortholog of S. cerevisiae/S. pombe Lsb5; predicted role in actin cortical patch localization, actin filament organization, endocytosis; flow model biofilm induced; Spider biofilm repressed
C3_01930W_A	PXP2	orf19.1655	Op	Op	Op	17.82	0.63	6.64	17	Uncharacterized ORF; Putative acyl-CoA oxidase; enzyme of fatty acid beta-oxidation; induced during macrophage infection; opaque specific transcript; putative peroxisome targeting signal; Spider biofilm induced
C7_02670W_A		orf19.3697				1.29	1.34	6.64	17	Uncharacterized ORF; Protein of unknown function
CR_03570C_A	YVH1	orf19.4401				0.94	1.2	6.64	17	Putative dual specificity phosphatase (phosphoserine/threonine and phosphotyrosine phosphatase); required for wild-type growth rate and for wild-type virulence in mouse model of systemic infection; Hap43p-induced gene
CR_01030W_A	DAL81	orf19.3252				0.93	0.83	6.64	17	Zn(II)2Cys6 transcription factor; ortholog of S. cerevisiae Dal81, involved in the regulation of nitrogen-degradation genes; required for yeast cell adherence to silicone substrate; Spider biofilm induced
C6_00310W_A		orf19.1191				0.78	0.85	6.64	17	Uncharacterized ORF; Protein of unknown function
C2_08280W_A	EAF3	orf19.2660				0.89	0.73	6.64	17	Subunit of the NuA4 histone acetyltransferase complex; rat catheter and Spider biofilm induced
C7_02560W_A		orf19.6453				1.14	1.24	6.64	17	Uncharacterized ORF; Protein of unknown function
C5_05170W_A	PAN3	orf19.4010				0.71	0.99	6.64	17	Uncharacterized ORF; Protein of unknown function
C7_03890C_A		orf19.7193				1.13	0.93	6.64	17	Uncharacterized ORF; Specificity factor required for ubiquitination; role in protein targeting to vacuole; involved in ubiquitin-dependent protein catabolism via the multivesicular body sorting pathway; Spider biofilm induced
CR_02790C_A	ALG5	orf19.2837	Op	Op	Op	1.04	0.55	6.64	17	Uncharacterized ORF; Putative glucosyltransferase involved in cell wall mannan biosynthesis; possibly an essential gene, disruptants not obtained by UAU1 method
C2_03120W_A	AMO1	orf19.5784				1.57	1.13	6.64	17	Uncharacterized ORF; Putative peroxisomal copper amine oxidase
CR_00330C_A	PXA1	orf19.7500				1.23	0.71	6.64	17	Uncharacterized ORF; Putative peroxisomal, half-size adrenoleukodystrophy protein (ALD or ALDp) subfamily ABC family transporter
C1_02110C_A	HGT2	orf19.3668		Op	Op	20.25	0.49	6.64	17	Putative MFS glucose transporter; 20 member C. albicans glucose transporter family; 12 probable membrane-spanning segments; expressed in rich medium with 2% glucose; rat catheter and Spider biofilm induced
C7_02350C_A		orf19.6476				1.44	1.4	6.64	17	Uncharacterized ORF; Putative protein with a predicted role in exocytic transport from the Golgi; filament induced

C2_09920W_A		orf19.1360				1.05	1.19	6.64	17	Uncharacterized ORF; Protein of unknown function
C5_04600C_A	GRR1	orf19.3944				0.8	0.79	6.64	17	F-box protein component of the SCF ubiquitin-ligase complex required for cell cycle progression; involved in negative control of pseudohyphal growth; Spider biofilm induced
C3_07680W_A		orf19.6719				0.93	0.96	6.64	17	Putative nucleosome assembly protein; homozygous transposon insertion causes decreased colony wrinkling under filamentous growth-inducing conditions, but does not block true hyphal formation in liquid media
C4_01940W_A	PHO89	orf19.4599	Op	Op	Op	0.86	0.69	6.64	17	Uncharacterized ORF; Putative phosphate permease; transcript regulated upon white-opaque switch; alkaline induced by Rim101; possibly adherence-induced; F-12/CO2 model, rat catheter and Spider biofilm induced
CR_03410W_A		orf19.2381				0.99	0.93	6.64	17	Uncharacterized ORF; Protein of unknown function; possibly an essential gene, disruptants not obtained by UAU1 method
C7_02850W_A		orf19.5177				1.13	1.07	6.64	17	Uncharacterized ORF; Protein of unknown function
C3_07710W_A	ABD1	orf19.6716				0.81	0.97	6.64	17	SAM-dependent RNA methyltransferase; methylates mRNA 5' cap; binds phosphorylated RNA Pol II C-term domain peptide; does not bind mRNA TPase and mRNA GTase (Cet1,Cgt1); functional homolog of S. cerevisiae Abd1; rat catheter biofilm induced
C1_04540C_A	VPS16	orf19.6848				1.15	1.32	6.64	17	Protein similar to S. cerevisiae Vps16p, which is involved in protein-vacuolar targeting; likely to be essential for growth, based on insertional mutagenesis; downregulated in biofilm or in azole-resistant strain that overexpresses MDR1
C1_05800C_A		orf19.2472				1.43	1.5	6.64	17	Uncharacterized ORF; Ortholog of S. pombe replication termination factor Rtf2; Spider biofilm induced
C5_02120C_A	RIX7	orf19.4219				0.82	1.12	6.64	17	Putative ATPase of the AAA family; role in ribosomal subunit export from the nucleus; mutation impairs hyphal growth and biofilm formation
CR_10290C_A		orf19.7604				0.82	0.83	6.64	17	Uncharacterized ORF; Protein of unknown function
C2_00730C_A		orf19.2051				0.76	0.86	6.64	17	Uncharacterized ORF; Protein of unknown function
C5_04840C_A		orf19.3970				1.09	1.2	6.64	17	Uncharacterized ORF; Putative ribosome biogenesis factor; possibly essential, disruptants not obtained by UAU1 method; rat catheter and Spider biofilm induced
C7_01500W_A		orf19.6579				1.32	1.113	6.64	17	Uncharacterized ORF; Protein of unknown function
C4_02930W_A		orf19.2701	Op	Op	Op	2.89	0.4	6.64	17	Uncharacterized ORF; Protein of unknown function; Ras1 and Cyr1 repressed; rat catheter and Spider biofilm induced
C7_00410C_A		orf19.7079				1.53	1.15	6.64	17	Uncharacterized ORF; Protein of unknown function
C1_11790W_A		orf19.1137				1.35	1.42	6.64	17	Uncharacterized ORF; Protein of unknown function
CR_01420W_A		orf19.2528				0.89	1.19	6.64	17	Uncharacterized ORF; Putative RNA polymerase III transcription factor (TFIIIB) subunit; flucytosine repressed
C6_03640W_A	NOG2	orf19.5732				0.83	1.09	6.64	17	Uncharacterized ORF; Putative nucleolar GTPase; repressed by prostaglandins; Hap43-induced, rat catheter and Spider biofilm induced
C2_06030W_A		orf19.4117				1.06	0.94	6.64	17	Uncharacterized ORF; Protein of unknown function
CR_07750C_A		orf19.3704				0.89	1.08	6.64	17	Uncharacterized ORF; Protein of unknown function
C3_06740W_A		orf19.6829				0.94	0.91	6.64	17	Uncharacterized ORF; Protein with a predicted mitochondrial ATPase expression domain; possibly an essential gene, disruptants not obtained by UAU1 method
C7_02010C_A		orf19.6518	Op	Op	Op	2.47	1.03	6.64	17	Uncharacterized ORF; Predicted aldehyde dehydrogenase [NAD(P)+]; Spider biofilm induced
C3_06400C_A		orf19.7425				0.97	0.75	6.64	17	Uncharacterized ORF; Protein of unknown function
C4_05590W_A		orf19.1240				1.05	1.05	6.64	17	Uncharacterized ORF; Protein of unknown function
C2_07160W_A	SMF12	orf19.2270				0.78	0.78	6.64	17	Uncharacterized ORF; Ortholog of S. cerevisiae Smf1; manganese transporter; Gcn4-regulated; Hap43, alkaline induced; caspofungin repressed; mutants are viable
C2_09630C_A		orf19.1391				1.09	0.99	6.64	17	Uncharacterized ORF; Protein with similarity to trimeric LpxA-like enzymes; Hap43-repressed gene
C6_04500C_A	TSM1	orf19.2135				0.97	0.96	6.64	17	Uncharacterized ORF; Putative transcription initiation factor TFIID subunit; transcript is upregulated in clinical isolates from HIV+ patients with oral candidiasis; Nrg1-regulated
CR_02250C_A	CEX1	orf19.3744				0.89	0.9	6.64	17	Uncharacterized ORF; Protein of unknown function
C5_02630C_A	MNN1	orf19.4279	Op		Op	13.35	0.95	6.64	17	Uncharacterized ORF; Putative alpha-1,3-mannosyltransferase; of the mannosyltransferase complex; negatively regulated by Rim101; transcript elevated in chk1 and nik1 mutants, but not in sln1 mutant; Spider and flow model biofilm induced
C4_04780W_A	PMT6	orf19.3802		Wh		0.75	0.9	6.64	17	Protein mannosyltransferase; required for virulence in mice, adhesion to endothelium; role in hyphal growth signaling, hygromycin B sensitivity; no major role in cellular PMT activity; Hap4-repressed; Bcr1-repressed in RPMI a/a biofilms
C1_04010C_A		orf19.4476		Op	Op	3.66	1.06	6.64	17	Uncharacterized ORF; Protein with a NADP-dependent oxidoreductase domain; transcript induced by ketoconazole; rat catheter and Spider biofilm induced
C1_12070C_A		orf19.5258				1.39	1.52	6.64	17	Uncharacterized ORF; Protein of unknown function; induced by nitric oxide
CR_01700C_A		orf19.2563				1.02	1.15	6.64	17	Uncharacterized ORF; Protein of unknown function
C1_04900W_A	MNN15	orf19.753				1.51	1.66	6.64	17	Uncharacterized ORF; Putative alpha-1,3-mannosyltransferase; predicted role in protein O-linked glycosylation; Spider biofilm induced
CR_03900W_A	HST7	orf19.469				0.84	1.09	6.64	17	MAP kinase kinase involved in mating and hyphal growth signal transduction pathways; phosphorylates Cek1p; wild-type virulence in mouse systemic infection; functional homolog of S. cerevisiae Ste7p; mutants are hypersensitive to caspofungin
C3_00490W_A	ESA1	orf19.5416				1.01	0.98	6.64	17	Subunit of the NuA4 histone acetyltransferase complex; member of MYST family; Spider biofilm induced
C4_07220C_A		orf19.3080				0.93	0.73	6.64	17	Uncharacterized ORF; Protein of unknown function
C1_01550W_A	VPS17	orf19.3344				1.37	1.33	6.64	17	Uncharacterized ORF; Protein of unknown function
C4_06450W_A		orf19.2889				1.05	0.97	6.64	17	Uncharacterized ORF; Protein of unknown function
CR_03100W_A	SMC5	orf19.2417				0.81	0.93	6.64	17	Protein similar to S. cerevisiae Smc5p, which is involved in DNA repair; transposon mutation affects filamentous growth
CR_10500C_A		orf19.7627				1.18	0.89	6.64	17	Uncharacterized ORF; Protein of unknown function
CM_00240W CM_00240	CGDID:CAL000	CaalfMp08.3						6.64	17	Uncharacterized ORF; Putative endonuclease encoded by the first three exons and part of the third intron of the mitochondrial COX1 gene; C-terminus contains a LAGLIDADG domain found in intron-encoded endonucleases; conserved in fungal mitochondria
C3_05460W_A		orf19.6984				0.89	1.04	6.64	17	Uncharacterized ORF; Protein of unknown function
C1_11090C_A		orf19.2307				1.37	1.58	6.64	17	Uncharacterized ORF; Protein of unknown function
CR_03200C_A		orf19.2404				0.85	1.11	6.64	17	Uncharacterized ORF; Protein of unknown function
C3_02560W_A	DDC1	orf19.245				0.95	1.05	6.64	17	Uncharacterized ORF; Putative DNA damage checkpoint protein; transcript induced during filamentous growth; regulated by Nrg1, Tup1
C6_00430C_A		orf19.1204				0.92	0.92	6.64	17	Uncharacterized ORF; Phosphorylated protein of unknown function; transcript is upregulated clinical isolates from HIV positive patients with oral candidiasis
C3_01540W_A		orf19.1691		Wh	Wh	0.21	0.67	6.64	17	Plasma-membrane-localized protein; filament induced; Hog1, ketoconazole, fluconazole and hypoxia-induced; regulated by Nrg1, Tup1, Upc2; induced by prostaglandins; flow model biofilm induced; rat catheter and Spider biofilm repressed
CR_06910W_A	BUD5	orf19.1842			Op	1.3	0.79	6.64	17	Predicted GTP/GDP exchange factor for Rsr1; rat catheter biofilm induced
C6_01110W_A	FAD2	orf19.118				0.78	0.88	6.64	17	Delta-12 fatty acid desaturase, involved in production of linoleic acid, which is a major component of membranes
C4_06230C_A		orf19.2915				1.08	0.95	6.64	17	Uncharacterized ORF; Protein of unknown function
C2_01920C_A		orf19.1504				1.13	1.17	6.64	17	Uncharacterized ORF; Putative patatin-like phospholipase; fungal-specific (no human or murine homolog)
C5_00780C_A		orf19.580				0.9	1.11	6.64	17	Uncharacterized ORF; Protein of unknown function; mutants are viable; Spider biofilm induced
C4_05790W_A		orf19.1265				0.83	0.93	6.64	17	Uncharacterized ORF; Protein of unknown function
C4_02830C_A	HCA4	orf19.2712				0.89	0.99	6.64	17	Uncharacterized ORF; Putative role in regulation of cell wall biogenesis; Hap43p-induced gene; possibly an essential gene, disruptants not obtained by UAU1 method; flow model and rat catheter biofilm induced
C3_05410W_A		orf19.6979				0.8	1.21	6.64	17	Uncharacterized ORF; Protein of unknown function
CR_03920C_A	TPO4	orf19.473	Wh	Wh		0.49	1.31	6.64	17	Uncharacterized ORF; Putative sperimidine transporter; fungal-specific (no human or murine homolog); Spider biofilm induced; promoter bound by Tec1 and Ndt80; Bcr1-repressed in RPMI a/a biofilms
C6_02960W_A		orf19.5574				0.96	1.21	6.64	17	Uncharacterized ORF; Protein of unknown function
C1_14230C_A	IRR1	orf19.7232				1.49	1.62	6.64	17	Uncharacterized ORF; Putative cohesin complex subunit; cell-cycle regulated periodic mRNA expression
C5_04940W_A		orf19.3982		Op		1.23	0.86	6.64	17	Maltase; induced during growth on sucrose; induced by alpha pheromone in SpiderM medium; early-stage flow model biofilm indced
C1_00130C_A	VPS53	orf19.6094				1.33	1.28	6.64	17	Uncharacterized ORF; Protein of unknown function
C4_00780C_A		orf19.4163				0.77	0.65	6.64	17	Uncharacterized ORF; Protein of unknown function
CR_06090W_A		orf19.3862				0.93	0.93	6.64	17	Uncharacterized ORF; Putative intracellular transport protein; heterozygous null mutant displays sensitivity to rapamycin; expression upregulated during growth in the mouse cecum
C1_13080W_A	OP4	orf19.4934	Op	Op		106.27	0.41	6.64	17	Ala- Leu- and Ser-rich protein; secreted; N-terminal hydrophobic region; possible glycosylation; opaque-specific transcript; repressed by alpha pheromone in opaque MTLa homozygotes; fluconazole-induced; Spider biofilm induced
C4_01360W_A	PGA53	orf19.4651		Op	Op	4.42	0.74	6.64	17	GPI-anchored cell surface protein of unknown function; greater mRNA abundance observed in a cyr1 homozygous null mutant than in wild type
C6_03780C_A		orf19.5752				1.31	0.92	6.64	17	Uncharacterized ORF; Protein of unknown function
CR_07310W_A		orf19.6137				0.87	1.06	6.64	17	Uncharacterized ORF; Protein of unknown function
C5_00980W_A	TRY3	orf19.1971			Wh	0.73	1.3	6.64	17	RING-finger transcription factor; regulator of yeast form adherence; required for yeast cell adherence to silicone substrate; Spider biofilm induced
C4_02300W_A	HOS3	orf19.2772			Wh	0.76	0.99	6.64	17	Uncharacterized ORF; Histone deacetylase; similar to S. cerevisiae Hos3p; greater expression and longer mRNA in white cells, compared to opaque cells; has conserved deacetylation motif
C7_02860C_A		orf19.5175				1.02	1.16	6.64	17	Uncharacterized ORF; Protein of unknown function
C2_09560C_A		orf19.1400				0.96	0.94	6.64	17	Uncharacterized ORF; Protein of unknown function
CR_01020C_A		orf19.3254				1.03	0.88	6.64	17	Uncharacterized ORF; Protein of unknown function
C1_04630C_A		orf19.783				1.47	1.39	6.64	17	Uncharacterized ORF; Protein of unknown function
C2_03800C_A		orf19.841				0.96	0.92	6.64	17	Uncharacterized ORF; Protein of unknown function
C4_02770C_A		orf19.2721				0.72	1	6.64	17	Uncharacterized ORF; Protein of unknown function
C3_07360W_A	DLD2	orf19.6755				0.8	0.58	6.64	17	Uncharacterized ORF; Protein of unknown function
C1_06440C_A		orf19.6266				1.11	1.16	6.64	17	Uncharacterized ORF; Protein of unknown function; Spider biofilm induced
CR_06450W_A		orf19.721				0.9	1.2	6.64	17	Uncharacterized ORF; Protein of unknown function
C1_05750C_A		orf19.2476				1.72	1.35	6.64	17	Uncharacterized ORF; Protein of unknown function
CR_00630W_A		orf19.7468				0.69	0.88	6.64	17	Uncharacterized ORF; Putative transcriptional activator; expression upregulated during growth in the mouse cecum; Spider biofilm induced
C1_01560W_A	SIZ1	orf19.3345				1.25	1.22	6.64	17	Uncharacterized ORF; Possible SUMO/Smt3 ligase; Rim101-repressed
C2_10140W_A		orf19.1767		Op		0.83	0.81	6.64	17	Uncharacterized ORF; Protein of unknown function
C1_03930W_A		orf19.4467				1.25	1.29	6.64	17	Uncharacterized ORF; Protein of unknown function
CR_07350W_A		orf19.6134				0.68	0.79	6.64	17	Uncharacterized ORF; Protein of unknown function
C1_13170C_A	MSH6	orf19.4945				1.49	1.53	6.64	17	Uncharacterized ORF; Protein similar to S. cerevisiae Msh6p, which is involved in mismatch repair; repressed under Cdc5p depletion; Hap43p-induced gene
CR_08070W_A	PTP1	orf19.6365				0.79	0.85	6.64	17	Uncharacterized ORF; Phosphotyrosine-specific protein phosphatase; rat catheter biofilm induced
C2_07470W_A	YCS4	orf19.1883				0.88	0.99	6.64	17	Uncharacterized ORF; Putative condensin complex subunit; cell-cycle regulated periodic mRNA expression
C2_05840W_A		orf19.5207				0.8	1.22	6.64	17	Uncharacterized ORF; Predicted diphthamide biosynthesis protein; Spider biofilm induced
C3_06310C_A	ISW2	orf19.7401				0.91	1.06	6.64	17	Ortholog of S. cerevisiae Isw2; an ATPase involved in chromatin remodeling; required for chlamydospore formation; Hap43-induced gene; repressed by high-level peroxide stress

C1_09390W_A		orf19.4801				1.19	1.22	6.64	17	Uncharacterized ORF; Protein of unknown function
CR_08650C_A		orf19.6440				1.01	0.85	6.64	17	Uncharacterized ORF; Protein of unknown function
C1_00360W_A	CDC13	orf19.6072				1.44	1.33	6.64	17	Essential protein with similarity to S. cerevisiae Cdc13p, involved in telomere maintenance
CR_07680C_A		orf19.6282			Op	1.22	0.45	6.64	17	Uncharacterized ORF; Protein of unknown function
C2_09500W_A		orf19.1404				1.06	1.09	6.64	17	Uncharacterized ORF; Predicted tRNA dihydrouridine synthase; Spider biofilm induced
C5_05210W_A		orf19.4014				1.17	0.73	6.64	17	Uncharacterized ORF; Planktonic growth-induced gene
C4_03720C_A		orf19.1306				0.95	0.95	6.64	17	Uncharacterized ORF; Protein of unknown function
C2_07090C_A	OLE2	orf19.2264				0.88	1.06	6.64	17	Protein with similarity to fatty acid desaturase (stearoyl-CoA desaturase); homozygous null mutant shows decreased production of prostaglandin E2
C5_02320C_A		orf19.4240				1.1	0.91	6.64	17	Uncharacterized ORF; Protein of unknown function
CR_08190W_A		orf19.6379				0.77	0.8	6.64	17	Uncharacterized ORF; Protein of unknown function
CR_06640C_A		orf19.698				0.92	0.8	6.64	17	Uncharacterized ORF; Integral ER membrane protein; predicted role in maintenance of ER zinc homeostasis; Spider biofilm induced
C1_02910C_A		orf19.2982				1.52	1.26	6.64	17	Uncharacterized ORF; Protein of unknown function
C6_00480C_A	FET31	orf19.4211		Op	op	1.75	0.6	6.64	17	Multicopper oxidase; for growth in low iron, prostaglandin E2 synthesis; ketoconazole/caspofungin/amphotericin B repressed; Sef1/Sfu1/Hap43 regulated; reports differ if functional homolog of ScFet3; rat catheter and Spider biofilm induced
C2_07810W_A		orf19.2200				1.13	0.89	6.64	17	Uncharacterized ORF; Protein of unknown function; rat catheter biofilm repressed
C5_04680W_A	PSD2	orf19.3954				0.58	0.67	6.64	17	Uncharacterized ORF; Protein of unknown function
C1_10560C_A		orf19.1002				1.37	0.93	6.64	17	Uncharacterized ORF; Protein of unknown function; Hap43-repressed gene
C1_00690W_A	RIC1	orf19.6036				1.15	1.17	6.64	17	Uncharacterized ORF; Ortholog of S. cerevisiae Ric1 guanyl-nucleotide exchange factor; mutant is viable; rat catheter biofilm repressed
CR_03260W_A		orf19.2397.3				0.85	0.69	6.64	17	Putative aminotransferase; Hap43-repressed; homozygous Tn insertion decreases colony wrinkling in filament-inducing conditions, does not block true hypha formation in liquid media; rat catheter and Spider biofilm induced
C3_05010C_A		orf19.5970				0.95	0.85	6.64	17	Uncharacterized ORF; Putative DNA repair helicase; transcriptionally induced by interaction with macrophage; fungal-specific (no human or murine homolog)
C5_00570W_A		orf19.932				0.71	1.22	6.64	17	Uncharacterized ORF; Putative aminophospholipid translocase (flippase); merged with orf19.2226 in Assembly 21; possibly an essential gene, disruptants not obtained by UAU1 method
C5_04190W_A	MRV2	orf19.3902	Op	Op	Op	7.63	0.08	6.64	17	Uncharacterized ORF; Protein of unknown function; repressed by fluphenazine treatment or in an azole-resistant strain that overexpresses CDR1 and CDR2; Spider biofilm induced
C6_00320C_A	DNA2	orf19.1192				0.91	0.85	6.64	17	Uncharacterized ORF; Protein similar to S. cerevisiae Dna2p, which is a DNA replication factor involved in DNA repair; induced under hydroxyurea treatment
C4_05090C_A	SRB8	orf19.736				0.91	0.92	6.64	17	Uncharacterized ORF; Putative RNA polymerase II mediator complex subunit; early-stage flow model biofilm induced
C2_07560W_A	SWR1	orf19.1871				0.88	0.91	6.64	17	Uncharacterized ORF; Protein of unknown function
C4_00590C_A		orf19.4184				0.84	0.86	6.64	17	Uncharacterized ORF; Protein with a predicted role in clathrin cage assembly; Hap43-repressed; Spider biofilm repressed
C5_02900W_A		orf19.4312				0.92	1.02	6.64	17	Uncharacterized ORF; Protein of unknown function
C1_02610W_A	IML1	orf19.2949				1.05	1.57	6.64	17	Uncharacterized ORF; Putative protein with a role in autophagy; rat catheter biofilm induced
C3_05850W_A		orf19.7365				0.92	0.92	6.64	17	Uncharacterized ORF; Protein of unknown function
C5_02180C_A	LEU3	orf19.4225				0.84	3.2	6.64	17	Zn(II)2Cys6 transcription factor; predicted regulator branched-c ofhain amino acid biosynthesis genes; alkaline induced; induced by Mnl1 under weak acid stress; required for yeast cell adherence to silicone substrate; Spider biofilm induced
C2_06320W_A		orf19.5495				0.69	0.94	6.64	17	Uncharacterized ORF; Putative RNA-binding protein; induced by alpha pheromone in SpiderM medium
C1_09280W_A		orf19.4787				1.32	1.25	6.64	17	Uncharacterized ORF; Protein of unknown function
C1_00870W_A	LRO1	orf19.6018				1.49	1.54	6.64	17	Uncharacterized ORF; Acyltransferase that catalyzes diacylglycerol esterification of phospholipids; role in lipid storage, triglyceride biosynthesis; flow model biofilm repressed
CR_08660W_A	PRP8	orf19.6442				0.94	1.05	6.64	17	Uncharacterized ORF; Protein similar to S. cerevisiae Prp8, a component of the U4/U6-U5 snRNP complex; repressed by alpha pheromone in SpiderM medium
C2_01400C_A	ESC4	orf19.1445				0.96	1.18	6.64	17	Protein similar to S. cerevisiae Esc4; a protein that represses transposition; transposon mutation affects filamentation; rat catheter biofilm repressed
C1_12600C_A	CHR1	orf19.3756				1.38	1.62	6.64	17	Uncharacterized ORF; Predicted DEAD-box ATP-dependent RNA helicase; functional homolog of S. cerevisiae Rok1; Hap43-induced; Spider biofilm induced
#N/D	NAD4	CaalfMp14				0.73	0.84	6.64	17	Uncharacterized ORF; Subunit 4 of NADH:ubiquinone oxidoreductase (NADH:ubiquinone dehydrogenase), a multisubunit enzyme complex (complex I) of the mitochondrial inner membrane that catalyzes the first step in mitochondrial respiration
C5_01840C_A	TAC1	orf19.3188				0.71	0.86	6.64	17	Zn(2)-Cys(6) transcriptional activator of drug-responsive genes (CDR1 and CDR2); binds DRE element; gene in zinc cluster region near MTL locus; resequencing indicates that TAC1 spans orf19.3188 and orf19.3189; Spider biofilm induced

Descriptions of published white (w) and opaque (o) gene sets refer to: [*] Lan et al. (2002) *PNAS* 23: 14902-12; [**] Tsong et al. (2003) *Cell* 115: 389-99; [21] Tuch et al. (2010) *PLoS Genetics* 6: e1001070; [34] Pande et al. (2013) *Nat. Gen* et., 45, 1088-1091.

URL. *Candida* Genome Database, http://www.candidagenome.org/.
